# Supplementary material for: Early Expression of Tet1 and Tet2 in Mouse Zygotes Altered DNA Methylation Status and Affected Embryonic Development
Source: Int J Mol Sci. 2022 Jul 31;23(15):8495. doi: 10.3390/ijms23158495 (PMC9369288; doi:10.3390/ijms23158495)
Supplement: Supplementary file 1 [file ijms-23-08495-s001.zip › ijms-1829847-supplementary.pdf]

Supplementary Materials

Early expression of Tet1 and Tet2 in mouse zygotes altered DNA methylation status and affected embryonic development

Authors: Qi Qi*, Qianqian Wang*, Kailing Liu, Jiangyue Bian, Zhixuan Yu and Jian Hou [#]

Description of Supplementary Files

Supplementary Figure S1: The expression profile of Tet family members in preimplantation mouse embryos

Supplementary Table S1: BS-conversion efficiency and mapping rates

Supplementary Table S2: The numbers of CpG sites at five methylation levels

Supplementary Table S3: The number of methylation-changed sites in the Tet1 and Tet2 groups among functional regions

Supplementary Table S4: The number of methylation-changed sites in the Tet1 and Tet2 groups among repeats

Supplementary Table S5: Location of known germline imprinting control regions and *P*-values in comparison of methylation levels between groups

Supplementary Table S6: The primers used for quantitative reverse transcription PCR (qRT-PCR) examination of Tet mRNA levels in mouse embryos

Supplementary Dataset 1: Genes with abnormal promoter methylation caused by Tet1 in zygotes

Supplementary Dataset 2: Genes with abnormal promoter methylation caused by Tet2 in zygotes

Figure S1: The expression profile of Tet family members in preimplantation mouse embryos. Quantitative reverse transcription PCR (qRT-PCR) was used to measure the expression levels of Tet family members at different stages during preimplantation. This expression profile demonstrated that only Tet3 was highly expressed in oocytes and zygotes, while Tet1 and Tet2 were expressed from 2-cell to blastocyst stages. All examinations were performed in triplicate. Primer sequences can be found in Supplementary Table S6. For data normalization, the expression of Gapdh was used as internal control. The relative expression was calculated using the 2^ΔCt method.

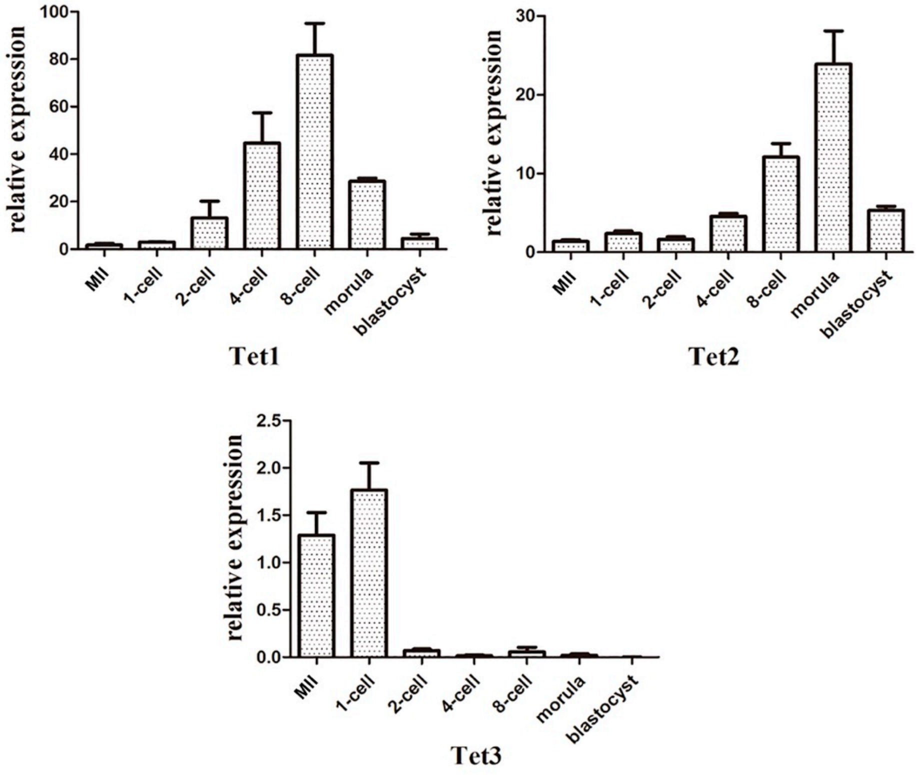

Table S1. BS-conversion efficiency and mapping rates. The BS conversion efficiency from 5 raw data is shown. The column for total reads shows the number of clean reads after data filtering. Mapped reads mean the number of reads that were uniquely mapped to the reference genome, and the unique mapping rates were calculated as mapped reads/total reads. The paternal and maternal genomes were split by using SNPs and their mapping rates are shown.

Samples	BS conversion rate(%)	Total reads	Mapped reads	Unique Mapping rate(%)
**sperm**	99.42	145433130	51500558	35.40
**Oocyte**	99.37	135097668	57677760	42.70
**GFP**	99.39	159473190	67672328	42.40
**GFP♀**		159473190	8988390	5.64
**GFP♂**		159473190	5237802	3.28
**Tet1**	99.43	176519558	69198774	39.20
**Tet1♀**		176519558	10156434	5.75
**Tet1♂**		176519558	3890382	2.20
**Tet2**	99.41	190224598	79390186	41.70
**Tet2♀**		190224598	7848576	4.13
**Tet2♂**		190224598	4670076	2.46

Table S2. The numbers of CpG sites at five methylation levels. Methylation levels (%) are indicated in the header of the table.

	0-20	20-40	40-60	60-80	80-100
**Sperm**	14004510	499963	1968441	1319939	48987932
**Oocyte**	18990869	194355	873044	149403	15355679
**GFP**	25501445	2376361	6923068	2748323	26970204
**GFP♀**	3352113	63143	326422	56947	2869481
**GFP♂**	2010312	17634	163869	23342	2048305
**Tet1**	27125231	2581426	7411877	2683736	23510164
**Tet1♀**	3273357	96590	502368	107480	3290409
**Tet1♂**	2087092	11134	96401	8464	1032037
**Tet2**	37947199	2395682	5226732	1168528	11889343
**Tet2♀**	3687250	63574	301857	42881	1689239
**Tet2♂**	3121416	13296	73316	6171	518335

Table S3. The number of methylation-changed sites among functional regions in the Tet1 and Tet2 groups. The methylation-changed sites in the Tet groups were determined by comparison with the GFP group, and the numbers of changed sites in the functional regions of the paternal genome and the maternal genome are shown, respectively. "↑" indicates hypermethylated sites, and "↓" indicates hypomethylated. The DMRs overlapping region >= 1bp were considered as an overlapped site listed in the rightmost column of each graph in a given context.

**Functional regions**	**♂ PN**			**♀ PN**		
	**Tet1 ♂**	**Tet2♂**	**Overlap**	**Tet1♀**	**Tet2♀**	**Overlap**
Promoter	3↑<br>33↓	0↑<br>140↓	0↑<br>13↓	85↑<br>28↓	25↑<br>100↓	3↑<br>3↓
5'UTR	1↑<br>10↓	0↑<br>55↓	0↑<br>5↓	20↑<br>8↓	4↑<br>40↓	1↑<br>0↓
Exon	12↑<br>344↓	0↑<br>1380↓	0↑<br>132↓	315↑<br>133↓	78↑<br>895↓	7↑<br>21↓
Intron	30↑<br>929↓	1↑<br>3906↓	0↑<br>309↓	1237↑<br>477↓	243↑<br>2771↓	21↑<br>82↓
3'UTR	3↑<br>65↓	0↑<br>244↓	0↑<br>28↓	58↑<br>29↓	11↑<br>174↓	1↑<br>5↓

Table S4. The number of methylation-changed sites among repeats in the Tet1 and Tet2 groups.

Repeats	♂ PN			♀ PN		
	Tet1♂	Tet2♂	Overlap	Tet♀	Tet2♀	Overlap
LINE	9↑<br>137↓	0↑<br>826↓	0↑<br>36↓	416↑<br>76↓	65↑<br>461↓	3↑<br>7↓
SINE	15↑<br>335↓	1↑<br>1686↓	0↑<br>88↓	833↑<br>182↓	159↑<br>1103↓	13↑<br>31↓
LTR	9↑<br>213↓	0↑<br>1182↓	0↑<br>55↓	677↑<br>108↓	124↑<br>735↓	15↑<br>17↓
DNA transposon	3↑<br>41↓	0↑<br>351↓	0↑<br>9↓	132↑<br>11↓	24↑<br>194↓	1↑<br>3↓
Low complexicy	2↑<br>15↓	0↑<br>134↓	0↑<br>3↓	38↑<br>9↓	4↑<br>67↓	0↑<br>0↓
Simple repeat	4↑<br>91↓	0↑<br>688↓	0↑<br>19↓	236↑<br>54↓	34↑<br>369↓	0↑<br>8↓

Table S5: Location of known germline imprinting control regions and *P*-values in comparison of methylation levels between groups. *P*-value<0.05 was considered significantly different.

Name	Status	Region			P-value		
		chr	start	end	GFPvsTet1	GFPvsTet2	Tet1 vsTet2
Mcts2/H13	M	2	152528194	152528847	1.0000000	0.8000280	0.6603591
Nespas/Gnasxl	M	2	174134696	174141579	0.4802875	0.0005328	0.0000361
Gnas1a	M	2	174168903	174169795	0.2444828	0.0000000	0.0002538
Peg10/Sgce	M	6	4746303	4749370	0.0000021	0.1236633	0.0015926
Mest_(Peg1)	M	6	30735839	30739964	0.0012832	0.0000673	0.0000000
Herc3/Nap1l5	M	6	58884386	58884779	0.6602285	0.6380090	1.0000000
Peg3/Usp29	M	7	6730355	6736208	0.9034564	0.0000026	0.0000038
Snurf/Snrpn	M	7	59652888	59655031	0.0010795	0.7889696	0.0247946
Kcnq1ot1	M	7	142848568	142849838	0.3288717	0.0090671	0.0000620
Plagl1	M	10	12965866	12968083	0.1702554	0.0059967	0.1610313
Grb10	M	11	11973322	11976797	0.0000004	0.0025261	0.0963086
Zrsr1/Commd1	M	11	22921545	22924145	0.0256836	0.0000000	0.0000090
Peg13/Trappc9	M	15	72680637	72683432	0.1114335	0.0605277	0.8592105
Slc38a4	M	15	96951881	96953622	0.2129026	0.0846712	0.7824123
Airn/Igf2r	M	17	12960647	12961836	0.0000018	0.6103762	0.0000159
Impact	M	18	13105904	13107805	0.0330959	0.0000000	0.0000004
H19_ICR	P	7	142133623	142135763	0.0516918	0.8102513	0.0507549
Rasgrf1	P	9	89751741	89761034	0.1059634	0.0065886	0.2540641
Dlk1-Gtl2_IG	P	12	109491681	109495763	0.2943442	0.4947330	0.7670240
H13_DMR2_(3'_end)	M	2	152549684	152550084	0.6611058	0.1805256	0.4481700
Casc1_intragenic	M	6	145133418	145133541	0.5883163	1.0000000	0.6685954
6330408a02Rik_3'_end	M	7	12995004	12995487	0.1347934	0.0002512	0.0500584
AK086712_promoter	M	7	61859003	61859312	1.0000000	0.5000000	0.4852941
FR149454_promoter	M	11	119149229	119150147	0.8777941	0.0066885	0.0044308
FR085584_promoter	M	12	80214947	80215771	0.6288505	0.0029124	0.0002569
Nhlrc1_downstream	M	13	47164286	47164405	0.4087735	0.6042478	1.0000000
Myo10_intragenic	M	15	25713910	25714249	0.0681618	0.0025878	0.7437651
Pvt1_promoter	M	15	61909019	61909179	0.1736116	0.0935052	0.5742987

Table S6: The primers used for quantitative reverse transcription PCR (qRT-PCR) examination of Tet mRNA expression in mouse embryos

Name	Primer sequences	
Tet1	Forward primer:	5’-CCATTCTCACAAGGACATTCACA- 3’
	Reverse primer:	5’-GCAGGACGTGGAGTTGTTCA- 3’
Tet2	Forward primer:	5’-GCCATTCTCAGGAGTCACTGC-3’
	Reverse primer:	5’-ACTTCTCGATTGTCTTCTCTATTGAGG-3
Tet3	Forward primer:	5’-GGTCACAGCCTGCATGGACT-3”
	Reverse primer:	5’-AGCGATTGTCTTCCTTGGTCAG-3’
GAPDH	Forward primer:	5’-CATGGCCTTCCGTGTTCCTA-3”
	Reverse primer:	5’-GCCTGCTTCACCACCTTCTT-3’

Supplementary Dataset 1: Genes with abnormal promoter methylation caused by Tet1 in zygotes							
Gene	Methylation levels of promoter		Dif	Gene	Methylation levels of promoter		Dif
	GFP group	Tet1 group			GFP group	Tet1 group	
Gm28239	0.24	0.67	hyper	Gm15666	0.35	0.97	hyper
Gm26145	0.09	0.57	hyper	Gm3942	0.1	0.57	hyper
Zap70	0.74	0.35	hypo	Gm4065	0.11	0.56	hyper
Nif3l1	0.88	0.39	hypo	4932442E05Rik	0.15	0.47	hyper
Gm4319	0.57	0.2	hypo	Gm19090	0.08	0.64	hyper
A630001G21Rik	0.7	0.08	hypo	Hmga2	0.22	0.02	hypo
Neu2	0.51	0.16	hypo	Avpr1a	0.23	0.71	hyper
Gm28722	0.93	0.47	hypo	Lrp1	0.53	0.18	hypo
Ube2f	0.7	0.27	hypo	Rdh16	0.73	0.04	hypo
Gm29480	0.7	0.34	hypo	Mir6915	0.81	0.45	hypo
AC164418.1	0.08	0.95	hyper	Gm33326	0.06	0.36	hyper
Mfsd4a	0.45	0.11	hypo	Gm5608	0.06	0.41	hyper
Etnk2	0.37	0.07	hypo	Gm45276	0.5	0.14	hypo
Gm38140	0.46	0.16	hypo	Tm2d2	0.21	0.05	hypo
Tuba5-ps	0.11	0.46	hyper	5430421F17Rik	0.03	0.39	hyper
Gm26568	0.18	0.5	hyper	Gm45305	0.07	0.51	hyper
Gm17781	0.17	0.45	hyper	Gm8291	0.09	0.78	hyper
1700019P21Rik	0.66	0.12	hypo	Gm2366	0.87	0.65	hypo
BC034090	0.57	0.25	hypo	Gm32052	0.06	0.55	hyper
Tex50	0.67	0.16	hypo	5330439A09Rik	0.64	0.03	hypo
Dnm3	0.76	0.2	hypo	Clcn3	0.31	0.1	hypo
Ddr2	0.07	0.48	hyper	Ell	0.2	0.03	hypo
Tstd1	0.8	0.51	hypo	Gdf15	0.04	0.4	hyper
Gm16564	0.48	0.14	hypo	Gm10654	0.2	0.71	hyper
Gm20305	0.89	0.09	hypo	Tmem221	0.48	0.14	hypo
D1Pas1	0.18	0.53	hyper	Gm10649	0.14	0.02	hypo
Gm2272	0.86	0.49	hypo	Lsm6	0.44	0.03	hypo
1700034H15Rik	0.46	0.19	hypo	Palm3	0.23	0.02	hypo
Fbh1	0.18	0.03	hypo	Cbln1	0.2	0.04	hypo
Gm37160	0.97	0.67	hypo	Acd	0.53	0.33	hypo
Gm13363	0.06	0.81	hyper	Exosc6	0.55	0.27	hypo
Potegl	0.05	0.52	hyper	Gm20406	0.7	0.33	hypo
Sohlh1	0.04	0.59	hyper	AC141881.6	0.6	0.08	hypo
Spaca9	0.59	0.24	hypo	Gm22109	1	0.16	hypo
Ak8	0.49	0.23	hypo	Gm45866	0.66	0.11	hypo
Gm13412	0.28	0.66	hyper	Gm21738	0.33	0.5	hyper

Lrsam1	0.34	0.12	hypo	Thrb	0.67	0.17	hypo
Snora65	0.24	0.07	hypo	Gm26898	0.03	0.83	hyper
Garnl3	0.57	0.12	hypo	Tasor	0.05	0.29	hyper
Rabgap1	0.37	0.07	hypo	Ncoa4	0.52	0.05	hypo
Gm13544	0.01	0.43	hyper	Gm48134	1	0.37	hypo
Slc25a12	0.58	0.05	hypo	Gm6616	0.01	0.12	hyper
Dlx2	0.25	0.04	hypo	Gm8518	0.13	0.71	hyper
Rapgef4	0.05	0.33	hyper	Gm16617	0.14	0.6	hyper
Gm13661	0.1	0.58	hyper	Gm6740	0.71	0	hypo
Cd82	0.17	0.79	hyper	Trav15d-1-dv6d-1	0.59	0	hypo
Gm13801	0	0.73	hyper	Cdh24	0.27	0.11	hypo
Gm10800	0.33	0.48	hyper	Irf9	0.84	0.47	hypo
Gm13920	0.75	0.26	hypo	Cbln3	0.22	0.01	hypo
Mtln	0.33	0.01	hypo	Fgf9	0.04	0.41	hyper
Bcl2l11	0.31	0.07	hypo	Mir6539	0.92	0.55	hypo
4930473A02Rik	0.4	0.05	hypo	Gm49417	0.76	0.34	hypo
Avp	0.59	0.17	hypo	Gm49295	0.19	0.92	hyper
Nnat	0.6	0.23	hypo	Lrch1	0.74	0.25	hypo
Ctnnbl1	0.13	0.71	hyper	Mycbp2	0.04	0.34	hyper
Gm14267	0.81	0.39	hypo	Gm33299	0.04	0.29	hyper
Gm14321	0.58	0.12	hypo	Gm5089	0.57	0.24	hypo
Gm14262	0.58	0.11	hypo	Gm10722	0.32	0.46	hyper
1700021F07Rik	0.17	0.5	hyper	Gm11168	0.36	0.5	hyper
4921531C22Rik	0.15	0.04	hypo	Gm10721	0.3	0.47	hyper
Mtg2	0.47	0.13	hypo	Gm10720	0.32	0.46	hyper
Rbm3os	0.76	0.45	hypo	Gm10719	0.32	0.46	hyper
Ftsj1	0.02	0.39	hyper	Gm10718	0.29	0.43	hyper
Gm6798	0.02	0.48	hyper	Gm10717	0.34	0.48	hyper
Pgrmc1	0.03	0.28	hyper	Gm17535	0.34	0.48	hyper
Ndufa1	0.04	0.26	hyper	Gm10715	0.36	0.49	hyper
Tenm1	0.01	0.27	hyper	Slc36a4	0.37	0.11	hypo
Rap2c	0.03	0.24	hyper	Gm24067	0.21	0.05	hypo
Magea14	0.04	0.55	hyper	Gm23008	0.66	0.35	hypo
Fhl1	0.01	0.23	hyper	Elof1	0.33	0.08	hypo
Gm14662	0.23	0.05	hypo	Ncapd3	0.29	0.05	hypo
Gabrq	0	0.33	hyper	Kcnj5	0.01	0.33	hyper
Pnma3	0.73	0.34	hypo	Gm48401	0.31	0.77	hyper
Xlr4d-ps	0.11	0.6	hyper	Gm48293	0.98	0.66	hypo
Fam220-ps	0.03	0.31	hyper	Gm16096	0.68	0.17	hypo
Apoo	0.06	0.29	hyper	AC158355.1	0.69	0.25	hypo
Snx12	0.18	0.75	hyper	Hinfp	0	0.23	hyper

Gm6275	0.94	0.57	hypo	Smad3	0.24	0.05	hypo
Mageh1	0.06	0.46	hyper	Spg21	0.69	0.29	hypo
Syap1	0.08	0.51	hyper	Ankdd1a	0.01	0.34	hyper
Ap1s2	0.01	0.18	hyper	AC158997.1	0.6	0.07	hypo
Hccs	0.04	0.33	hyper	Senp6	0.54	0.08	hypo
Gm15247	0.64	0.32	hypo	Plscr2	0.89	0.3	hypo
Gm37459	0.07	0.48	hyper	Chst2	0.12	0.01	hypo
Gm10728	0.09	0.43	hyper	Clstn2	0	0.16	hyper
Gm43548	0	0.5	hyper	Gm37917	0.91	0.39	hypo
Plch1	0.77	0.08	hypo	5830462I19Rik	0.85	0.53	hypo
Ssr3	0.06	0.25	hyper	Ccdc12	0.11	0.43	hyper
B3galnt1	0.49	0.12	hypo	Dync1li1	0.05	0.22	hyper
Gm37933	0.2	0.07	hypo	Gm38642	0.06	0.38	hyper
Scamp3	0.74	0.39	hypo	Trak1	0.1	0.37	hyper
Efna1	0.38	0.09	hypo	Exosc7	0.23	0.03	hypo
Mir7014	0.83	0.48	hypo	Emid1	0.07	0.36	hyper
Gm17690	0.48	0.17	hypo	Snhg15	0.08	0.36	hyper
5730437C11Rik	0.64	0.16	hypo	2610001A08Rik	0.05	0.31	hyper
Wnt2b	0.37	0.09	hypo	Gm16140	0.04	0.3	hyper
Gstm4	0.04	0.42	hyper	Zfp2	0.1	0.52	hyper
Pla2g12a	0.33	0.08	hypo	Gm12226	0.84	0.32	hypo
Etnppl	0.84	0.32	hypo	Fam114a2	0.02	0.26	hyper
5830437K03Rik	0.02	0.29	hyper	Gm12246	0.79	0.31	hypo
Papss1	0	0.21	hyper	Gm12570	0.08	0.71	hyper
Tgs1	0.34	0.03	hypo	Trim17	0.13	0.01	hypo
Gm24016	0.26	0.06	hypo	H2aw	0.13	0.01	hypo
Klhl32	0.57	0.12	hypo	2610507I01Rik	0.55	0.24	hypo
Gm11918	0.17	0.73	hyper	Usp22	0.38	0.06	hypo
Lyrm2	0.02	0.43	hyper	Hs3st3a1	0.8	0.16	hypo
Gm12390	0.96	0.3	hypo	Efnb3	0.67	0.3	hypo
Gm12401	0.97	0.65	hypo	Mpdu1	0.67	0.34	hypo
Sigmar1	0.83	0.35	hypo	Mir1934	0.73	0.16	hypo
Pax5	0.18	0.01	hypo	4930563E22Rik	0.2	0.02	hypo
Gm12512	0.22	0.82	hyper	Sgsm2	0.42	0.08	hypo
Ifnz	0.04	0.18	hyper	Tsr1	0.19	0.06	hypo
Gm12692	0	0.46	hyper	Rffl	0.57	0.25	hypo
Foxd3	0.01	0.15	hyper	Dgkeos	0.08	0.54	hyper
Usp24	0.26	0.05	hypo	Hoxb5	0.06	0.26	hyper
AL627238.2	0.13	0.72	hyper	4933428G20Rik	0.03	0.15	hyper
Gm12867	0.78	0.45	hypo	Gm12352	0.89	0.43	hypo
9930104L06Rik	0.04	0.2	hyper	Gm12359	0.64	0.17	hypo
Gm13257	0.66	0.16	hypo	Cavin1	0.11	0.67	hyper

Rap1gap	0.76	0.23	hypo	Psmc3ip	0.09	0.61	hyper
Mrto4	0.4	0.14	hypo	Plekhh3	0.3	0.05	hypo
Efhd2	0.04	0.23	hyper	Higd1b	0.95	0.45	hypo
Tnfrsf8	0.71	0.28	hypo	Arhgap27os3	0.04	0.52	hyper
Smarca5-ps	0.02	0.17	hyper	Limd2	0.77	0.47	hypo
Gm13231	0.03	0.28	hyper	4732490B19Rik	0.94	0.54	hypo
Gm13034	0.03	0.18	hyper	Gm11736	0.2	0.71	hyper
Gm13146	0.45	0.1	hypo	Gm11724	0.13	0.39	hyper
Gm13136	0.04	0.19	hyper	Gm11726	0.17	0.58	hyper
Mthfr	0.2	0.02	hypo	Myadml2os	0.38	0.66	hyper
Gm9506	0.97	0.47	hypo	Ptchd3	0.1	0.7	hyper
Gm13091	0.67	0.23	hypo	Gm48544	0.59	0	hypo
Gm13090	0.86	0.44	hypo	H2bc6	0.07	0.38	hyper
Rnf207	0.1	0.49	hyper	Gm11398	0.07	0.38	hyper
Gm10564	0.8	0.49	hypo	H2ac1	0.09	0.33	hyper
Rbm48	0.39	0.08	hypo	Tubb2b	0.38	0.15	hypo
Gm43540	0.38	0.89	hyper	Gm26877	0.27	0.02	hypo
Gm43391	0.2	0.43	hyper	4930471G24Rik	0.51	0.13	hypo
mmu-mir-12197	0.24	0.56	hyper	Gm47071	0.61	0.34	hypo
Psmc2	0.2	0.03	hypo	Gm17878	0.77	0.38	hypo
4632411P08Rik	0.31	0.13	hypo	Gm34788	0.13	0.71	hyper
Gm9924	0.25	0.04	hypo	Gm47585	0.55	0.22	hypo
Mpv17	0.59	0.19	hypo	Gm47602	0.89	0.35	hypo
Grk4	0.2	0.05	hypo	Gm10775	0.51	0.24	hypo
4930442P19Rik	0.13	0.03	hypo	Mtrr	0.24	0.05	hypo
Tada2b	0.23	0.05	hypo	Jmy	0.26	0	hypo
Hs3st1	0	0.61	hyper	Gm15620	0	0.38	hyper
Gm42646	0.08	0.73	hyper	Crhbp	0.16	0.58	hyper
Gm43004	0.11	0.53	hyper	Sv2c	0.22	0.04	hypo
Gm5867	0.1	0.69	hyper	Gfm2	0.39	0.09	hypo
Zar1	0.02	0.22	hyper	Gm47057	0.61	0.08	hypo
1700071G01Rik	0.67	0.24	hypo	Gm49496	0.1	0.49	hyper
4930467D21Rik	0.08	0.38	hyper	Gm15290	0.1	0.49	hyper
4930458D05Rik	0.2	0.63	hyper	Dhx29	0.31	0.07	hypo
Gm10359	0.06	0.57	hyper	Gm18244	0.95	0.43	hypo
Gm43818	0.11	0.64	hyper	Ncoa1	0	0.16	hyper
Fgfrl1	0.74	0.27	hypo	Pfn4	0.34	0.08	hypo
Gm43137	0.25	0.05	hypo	Nfyc-ps	0.04	0.52	hyper
Gm16019	0.93	0.41	hypo	4921511I17Rik	0	0.31	hyper
4933415J04Rik	0.2	0.7	hyper	Gm48140	0.91	0.48	hypo
F830115B05Rik	0.87	0.38	hypo	Gm9229	0.04	0.35	hyper
Gm42161	0.75	0.39	hypo	Gm49370	0.08	0.25	hyper

Myo1h	0.45	0.1	hypo	Gm18026	0.1	0.94	hyper
Gm13822	0.83	0.43	hypo	Sstr1	0.01	0.3	hyper
Gm42903	0.19	0.06	hypo	Gm47647	0	0.27	hyper
Gm42550	0.87	0.34	hypo	Trmt5	0.26	0.08	hypo
Kdm2b	0.72	0.18	hypo	Gm48388	0.18	0.63	hyper
A930024E05Rik	0.62	0.1	hypo	Gm16876	0.54	0.22	hypo
Gm43661	0.8	0.43	hypo	4930559C10Rik	0.17	0.61	hyper
Gm16001	0.5	0.25	hypo	Gm26839	0.14	0.02	hypo
Ogfod2	0.53	0.23	hypo	Ppp4r3a	0.43	0.09	hypo
Gm42885	0.24	0.76	hyper	Tunar	0.13	0.54	hyper
Dnajc30	0.4	0.12	hypo	Mir3544	0.89	0.57	hypo
6330403L08Rik	0.28	0.12	hypo	Mir433	0.95	0.7	hypo
Mir7037	0.92	0.69	hypo	Gm49090	0.2	0.58	hyper
Ttyh3	0.03	0.21	hyper	Siah1-ps2	0.31	0.68	hyper
Gm20635	0.26	0.08	hypo	Gm4740	0.03	0.44	hyper
Trrap	0.02	0.29	hyper	Gm37042	0.3	0.02	hypo
Gm42883	0.89	0.15	hypo	Scrib	0.67	0.36	hypo
Sgce	0.51	0.24	hypo	Gpt	0.83	0.52	hypo
Gm20619	0.1	0.45	hyper	Txn2	0.5	0.13	hypo
Gm16055	0.24	0.67	hyper	Tst	0.04	0.23	hyper
Gm30270	0.32	0.11	hypo	Gm10863	0.09	0.49	hyper
Lrrc4	0.58	0.1	hypo	Mir33	0.81	0.44	hypo
Smkr-ps	0.74	0.32	hypo	Rnu12	0.2	0.04	hypo
Chchd3	0.07	0.64	hyper	mmu-mir-12186	0.92	0.5	hypo
Fam180a	0.05	0.41	hyper	Gm49513	0.23	0.74	hyper
Gimap1os	0.31	0.06	hypo	Samm50	0.62	0.29	hypo
Evx1os	0.59	0.12	hypo	Nup50	0.37	0.08	hypo
Gm16499	0.24	0.07	hypo	Gm4825	0.23	0.65	hyper
Immt	0	0.23	hyper	Gm49464	0.07	0.4	hyper
Dnah6	0.53	0.13	hypo	Slc2a13	0	0.24	hyper
B230319C09Rik	0.67	0.38	hypo	Gm41386	0.19	0.7	hyper
1810020O05Rik	0.08	0.49	hyper	Gm49443	0.92	0.51	hypo
Gm20426	0.01	0.3	hyper	Rhebl1	0.04	0.2	hyper
Fgd5	0.32	0.04	hypo	Cox14	0.25	0.05	hypo
Gpr27	0.2	0.05	hypo	Tns2	0.64	0.31	hypo
Gm4875	0.2	0.55	hyper	Gm49477	0.89	0.41	hypo
Rps27a-ps3	0.92	0.54	hypo	Ercc4	0.62	0.17	hypo
Gm16557	0.67	0.12	hypo	Mir130b	0.43	0.13	hypo
1700027F06Rik	0.15	0.64	hyper	Mir301b	0.45	0.11	hypo
2010008C14Rik	0.1	0.25	hyper	Tango2	0.24	0.04	hypo
Tuba3a	0.79	0.41	hypo	Gp1bb	0.45	0.12	hypo
Lockd	0.16	0.03	hypo	Fetub	0.15	0.63	hyper

Gm23498	0.15	0.55	hyper	Atp13a4	0.26	0.7	hyper
Gm23462	0.97	0.55	hypo	Wdr5b	0.09	0.53	hyper
Gm15494	0.79	0.43	hypo	Trmt10c	0.32	0.65	hyper
Zfp264	0.06	0.42	hyper	Olfr201	0.06	0.68	hyper
Gm30645	0.9	0.27	hypo	Gm9843	0.92	0.53	hypo
Gm34779	0.03	0.58	hyper	Gm49961	0.2	0.65	hyper
Rsph6a	0.47	0.75	hyper	Tmem181b-ps	0.75	0.46	hypo
Gm26852	0.74	0.46	hypo	C87487	0.22	0.75	hyper
Ceacam20	0.21	0.56	hyper	Map3k4	0.43	0.05	hypo
Rps19	0.06	0.34	hyper	Gm49954	0.17	0.94	hyper
Megf8	0.18	0.02	hypo	Paqr4	0.09	0.26	hyper
Gm26604	0.21	0.51	hyper	9530082P21Rik	0.1	0.28	hyper
Zfp940	0.18	0.81	hyper	Arhgdig	0.25	0.65	hyper
Gm12756	0.01	0.15	hyper	Gm41555	0.65	0.17	hypo
Wdr88	0.94	0.46	hypo	Mir6968	0.46	0.1	hypo
Gm6004	0.06	0.81	hyper	Gm49789	0.87	0.42	hypo
Gm15545	0.31	0.05	hypo	AC174471.1	0.89	0.49	hypo
Snord35b	0.29	0.12	hypo	A530088E08Rik	0.07	0.24	hyper
Ruvbl2	0.75	0.25	hypo	Mir219c	0.22	0.1	hypo
Plekha4	0.42	0.11	hypo	Gm20460	0.49	0.11	hypo
Fgf21	0.14	0.44	hyper	Prrt1	0.52	0.09	hypo
Dbp	0.12	0.44	hyper	Fkbpl	0.05	0.38	hyper
Rpl18	0.65	0.31	hypo	Dxo	0.17	0.02	hypo
Kcnj11	0.15	0.63	hyper	Ly6g5b	0.66	0.19	hypo
Tm2d3	0.85	0.35	hypo	Mir6975	0.35	0.12	hypo
Mef2a	0.15	0.01	hypo	Mrpl2	0.29	0.03	hypo
Gm44669	0.87	0.53	hypo	Foxp4	0.55	0.13	hypo
Gm45206	0.68	0.35	hypo	Mir6977	0.95	0.73	hypo
Ap3b2	0.17	0.48	hyper	Gm46575	0.72	0.26	hypo
Tlnrd1	0	0.25	hyper	Txndc2	0.92	0.5	hypo
Gm15635	0.83	0.45	hypo	Xdh	0.82	0.15	hypo
Gm45667	0.02	0.16	hyper	Gm9386	0.21	0.67	hyper
4930560O18Rik	0.83	0.22	hypo	Gm26612	0.38	0.8	hyper
Abca15	0.03	0.28	hyper	Gm10308	0.02	0.46	hyper
Asphd1	0.56	0.1	hypo	Gm49990	0.03	0.53	hyper
Sez6l2	0.62	0.15	hypo	Gm9993	0.15	0.02	hypo
Maz	0.52	0.22	hypo	Gm49968	0.88	0.5	hypo
Gm49388	0.8	0.39	hypo	B930094E09Rik	0.56	0.15	hypo
9130023H24Rik	0.3	0.05	hypo	Epb41l4a	0.38	0.11	hypo
Gm43580	0.76	0.36	hypo	Gm10545	0.08	0.21	hyper
Gm44647	0.18	0.04	hypo	Gm26672	0.12	0.45	hyper
Mir210	0.53	0.22	hypo	Pcdhgb7	0.08	0.45	hyper

Gm25515	0.9	0.6	hypo	Gm38182	0.02	0.17	hyper
Armt1	0.36	0.07	hypo	Ticam2	0.04	0.43	hyper
Gm8330	0.23	0.72	hyper	Csnk1g3	0.25	0.05	hypo
Nhsl1	0.21	0.07	hypo	Mir6982	0.24	0.63	hyper
Gm49353	0.07	0.33	hyper	Gm9926	0.16	0.59	hyper
Gm9068	0.93	0.43	hypo	Gm5509	0.76	0.33	hypo
Ascc1	0.05	0.38	hyper	Gm41787	0.07	0.36	hyper
Gm47595	0.61	0.25	hypo	Gm7569	0.12	0.64	hyper
Gm16220	0.86	0.43	hypo	Gm50415	0.13	0.62	hyper
Vmn2r82	0.13	0.61	hyper	Cd248	0.42	0.09	hypo
Gzmm	0.95	0.65	hypo	Gm50208	0.45	0.77	hyper
2310011J03Rik	0.16	0.53	hyper	Gm6293	0.73	0.17	hypo
Mir1982	0.22	0.05	hypo	Gm16538	0.67	0.31	hypo
Snord37	0.4	0.16	hypo	Slc22a12	0.8	0.4	hypo
4930442H23Rik	0.83	0.34	hypo	AC109619.1	0.99	0.62	hypo
Mir3057	0.94	0.61	hypo	Fads3	0.73	0.19	hypo
Gm16315	0.76	0.39	hypo	Ms4a6c	0.21	0.86	hyper
4930404N11Rik	0.9	0.55	hypo	Gm50373	0.18	0.04	hypo
Gm16104	0.62	0.26	hypo	Gm50323	0.51	0.17	hypo
1500009L16Rik	0.01	0.28	hyper	Gm28578	0.15	0.66	hyper
Gm16270	0.12	0.48	hyper	Gm50431	0.78	0.39	hypo
4930555G07Rik	0.09	0.72	hyper	Gm10197	0.75	0.26	hypo
Gm35206	0.12	0.44	hyper	Gm50357	0.16	0.55	hyper

Supplementary Dataset 2: Genes with abnormal promoter methylation caused by Tet2 in zygotes							
Gene	Methylation levels of promoter		Dif	Gene	Methylation levels of promoter		Dif
	GFP group	Tet2 group			GFP group	Tet2 group	
Gm37381	0.66	0.32	hypo	Gm16230	0.23	0.02	hypo
Gm6104	0.76	0.26	hypo	Rdh16	0.73	0.00	hypo
Gm38376	0.80	0.42	hypo	Mir677	0.22	0.04	hypo
Gm38223	0.85	0.09	hypo	Gm23182	0.81	0.14	hypo
Ube2w	0.55	0.12	hypo	Gm17201	0.66	0.10	hypo
Col9a1	0.02	0.41	hyper	Slc39a5	0.61	0.05	hypo
Gm17813	0.86	0.12	hypo	Gm26347	0.81	0.30	hypo
1700001G17Rik	0.35	0.00	hypo	Smarcc2	0.30	0.11	hypo
Gm37354	0.73	0.17	hypo	Gm6336	0.93	0.10	hypo
Gm28631	0.59	0.19	hypo	Gm47093	0.73	0.28	hypo
Gm26788	0.55	0.28	hypo	Dnajc14	0.61	0.24	hypo
A930005N03Rik	0.70	0.25	hypo	Sarnp	0.71	0.13	hypo
Gm37958	0.50	0.06	hypo	Pcp2	0.59	0.15	hypo
Gm3052	0.96	0.48	hypo	Gm7389	0.50	0.05	hypo
Gm28306	0.87	0.36	hypo	Gpi-ps	0.85	0.19	hypo
Gm37146	0.59	0.11	hypo	Gm44515	0.91	0.48	hypo
Gm37909	0.34	0.08	hypo	4930453L07Rik	0.79	0.13	hypo
Gm42417	0.75	0.32	hypo	Gm44541	0.80	0.23	hypo
Gm33533	0.65	0.12	hypo	Gm44797	0.85	0.19	hypo
Gm37506	0.84	0.35	hypo	Gm15418	0.29	0.01	hypo
Gm38115	0.85	0.19	hypo	Gm18991	0.79	0.19	hypo
Gm37135	0.92	0.22	hypo	Tex29	0.83	0.51	hypo
4930556I23Rik	0.54	0.14	hypo	Mir1968	0.75	0.32	hypo
Gm16152	0.50	0.12	hypo	Atp4b	0.68	0.14	hypo
Gm15832	0.85	0.26	hypo	Gm26184	0.79	0.12	hypo
Gm37435	0.93	0.28	hypo	Gm45271	0.81	0.33	hypo
Slc9a2	0.74	0.05	hypo	Gm35453	0.76	0.18	hypo
Gm37623	0.91	0.12	hypo	Mir3106	0.79	0.16	hypo
Gm37047	0.44	0.10	hypo	Defb7	0.81	0.33	hypo
Gm16103	0.78	0.11	hypo	Gm7760	0.30	0.02	hypo
Gm37242	0.76	0.04	hypo	Gm10060	0.27	0.12	hypo
Gm28777	1.00	0.36	hypo	Gm7807	0.42	0.14	hypo
Gm6801	0.77	0.41	hypo	Gm21769	0.36	0.18	hypo
A130048G24Rik	0.96	0.27	hypo	Gm21112	0.37	0.14	hypo
Nif3l1	0.88	0.32	hypo	Defb35	0.72	0.25	hypo
Gm29017	0.84	0.31	hypo	1700041G16Rik	0.26	0.02	hypo
Gm26813	0.78	0.27	hypo	Gm45555	0.65	0.20	hypo

Gm11609	0.73	0.08	hypo	Gm26714	0.53	0.10	hypo
Gm11588	0.92	0.10	hypo	Tm2d2	0.21	0.03	hypo
Platr12	0.69	0.05	hypo	Mir8108	0.74	0.17	hypo
Mir6899	0.88	0.34	hypo	Plpp5	0.51	0.09	hypo
Gm13748	0.86	0.21	hypo	Gm45411	0.95	0.14	hypo
Gm28845	0.96	0.47	hypo	Gm32050	0.38	0.00	hypo
Spag16	0.50	0.12	hypo	Gm45572	0.66	0.07	hypo
4933417E11Rik	0.70	0.16	hypo	Gm45371	0.32	0.00	hypo
Gm5528	0.81	0.33	hypo	4933416M07Rik	0.59	0.08	hypo
Gm15843	0.85	0.09	hypo	Gm45304	0.93	0.00	hypo
Gm28364	0.74	0.39	hypo	Gm45817	0.76	0.25	hypo
Pnkd	0.18	0.01	hypo	Gm6100	0.45	0.04	hypo
Slc11a1	0.67	0.18	hypo	Gm33968	0.86	0.20	hypo
Mir26b	0.66	0.35	hypo	Gm34474	0.79	0.30	hypo
Ttll4	0.73	0.14	hypo	Gm19410	0.70	0.22	hypo
Gm29253	0.80	0.24	hypo	5430403N17Rik	0.93	0.20	hypo
Atg9a	0.16	0.01	hypo	Gm2366	0.87	0.47	hypo
Dnpep	0.47	0.11	hypo	Cfap97	0.24	0.02	hypo
Gm28410	0.47	0.00	hypo	Acsl1	0.35	0.08	hypo
A530032D15Rik	0.51	0.13	hypo	E030037K01Rik	0.56	0.20	hypo
Gm16028	0.83	0.49	hypo	Cldn24	0.48	0.06	hypo
Gm16025	0.50	0.11	hypo	Gm2607	0.47	0.10	hypo
Gm37914	0.87	0.28	hypo	Gm32842	0.78	0.25	hypo
Gm28626	0.71	0.37	hypo	Gm45830	0.76	0.26	hypo
Chrnd	0.52	0.02	hypo	Gm6012	0.85	0.19	hypo
Snorc	0.74	0.14	hypo	Gm45448	0.73	0.18	hypo
Gm52972	0.87	0.31	hypo	Nat1	0.61	0.08	hypo
Gm37521	0.80	0.24	hypo	Cers1	0.49	0.13	hypo
Gm28722	0.93	0.25	hypo	Rex1bd	0.70	0.14	hypo
4930474B08Rik	0.71	0.15	hypo	Uba52	0.45	0.07	hypo
Ube2f	0.70	0.35	hypo	Ell	0.20	0.02	hypo
Gm37048	0.70	0.20	hypo	Isyna1	0.35	0.07	hypo
Gm29481	0.79	0.30	hypo	Ifi30	0.53	0.22	hypo
Gm29480	0.70	0.28	hypo	Slc5a5	0.29	0.06	hypo
Gm28086	0.48	0.04	hypo	Mir7067	0.30	0.09	hypo
Mab21l4	0.41	0.10	hypo	Snora68	0.20	0.04	hypo
Gm17415	0.74	0.22	hypo	Mir7068	0.81	0.23	hypo
Bok	0.44	0.09	hypo	Gm45407	0.84	0.32	hypo
Atg4b	0.76	0.10	hypo	1700092C02Rik	0.79	0.07	hypo
Bcl2	0.19	0.00	hypo	D830024N08Rik	0.95	0.40	hypo
Mir3473f	0.47	0.02	hypo	Mir8111	0.51	0.09	hypo
Gm26080	0.74	0.13	hypo	Mir181d	0.71	0.08	hypo

Gm23497	0.88	0.24	hypo	Mir181c	0.67	0.08	hypo
3830432H09Rik	0.80	0.10	hypo	Mri1	0.37	0.06	hypo
Dbi	0.35	0.04	hypo	Mir7069	0.85	0.11	hypo
Gm37625	0.91	0.44	hypo	Rnaseh2a	0.16	0.01	hypo
Gm28914	0.32	0.00	hypo	Gm25506	0.93	0.17	hypo
Mfsd4a	0.45	0.12	hypo	Wdr83os	0.19	0.02	hypo
Cdk18	0.44	0.02	hypo	Orc6	0.20	0.02	hypo
Klhdc8a	0.36	0.01	hypo	4930535O05Rik	0.83	0.28	hypo
Gm10538	0.37	0.00	hypo	Gm45496	0.74	0.05	hypo
Ppfia4	0.83	0.19	hypo	Gm45293	0.76	0.36	hypo
Gm10535	0.71	0.19	hypo	Gm45804	0.67	0.14	hypo
Gpr37l1	0.55	0.10	hypo	Adgrg1	0.92	0.25	hypo
Gm29486	1.00	0.43	hypo	Gm45757	0.68	0.11	hypo
Gm37799	0.78	0.25	hypo	Gm45758	0.86	0.32	hypo
Gm33994	0.68	0.11	hypo	Gm31224	0.78	0.26	hypo
Gm37101	0.93	0.29	hypo	Gm45812	0.69	0.11	hypo
1700019P21Rik	0.66	0.11	hypo	Gm10631	0.55	0.07	hypo
Gm37423	0.83	0.09	hypo	Gm45750	0.75	0.35	hypo
Gm15479	0.74	0.20	hypo	Gm33023	0.33	0.03	hypo
Gm8976	0.52	0.07	hypo	Elmo3	0.66	0.12	hypo
BC034090	0.57	0.10	hypo	Mir328	0.80	0.39	hypo
Qsox1	0.29	0.06	hypo	Acd	0.53	0.18	hypo
Gm10531	0.81	0.29	hypo	Nutf2	0.29	0.02	hypo
Gm38256	0.68	0.07	hypo	Gm16156	0.69	0.21	hypo
Gm37848	0.81	0.17	hypo	6030452D12Rik	0.81	0.23	hypo
Gm36975	0.89	0.17	hypo	Pdf	0.62	0.14	hypo
Tex50	0.67	0.04	hypo	Lncbate1	0.93	0.41	hypo
Gm16588	0.77	0.06	hypo	Gm38042	0.81	0.21	hypo
Gm33354	0.88	0.20	hypo	Gm20686	0.79	0.39	hypo
Aldh9a1	0.40	0.00	hypo	Pmfbp1	0.81	0.15	hypo
Lrrc52	0.73	0.17	hypo	Zfp612	0.23	0.00	hypo
AC113490.1	0.85	0.34	hypo	Gm53030	0.66	0.20	hypo
Ndufs2	0.67	0.22	hypo	Pdpr	0.17	0.00	hypo
Arhgap30	0.52	0.07	hypo	Gm6793	0.83	0.25	hypo
Tstd1	0.80	0.36	hypo	AC114648.1	0.82	0.24	hypo
Gm37787	0.89	0.22	hypo	Syce1l	0.76	0.14	hypo
Gm17224	0.77	0.19	hypo	Clec3a	0.63	0.09	hypo
Igsf9	0.61	0.19	hypo	1700018P08Rik	0.95	0.35	hypo
Tagln2	0.61	0.15	hypo	Sdr42e1	0.46	0.00	hypo
Kmo	0.89	0.21	hypo	Gm45721	0.80	0.29	hypo
AC122854.1	0.98	0.55	hypo	Osgin1	0.72	0.25	hypo
Gm38053	0.94	0.41	hypo	Necab2	0.67	0.30	hypo

Gm37246	0.75	0.12	hypo	Wfdc1	0.71	0.11	hypo
Gm15423	0.63	0.14	hypo	Gm26537	0.63	0.17	hypo
2310043L19Rik	0.76	0.31	hypo	Gm26784	0.57	0.26	hypo
Gm8010	0.87	0.00	hypo	Gm20406	0.70	0.12	hypo
Gm16564	0.48	0.16	hypo	Gm17709	0.77	0.44	hypo
Gm37267	0.81	0.19	hypo	Mir7237	0.79	0.35	hypo
Gm24836	0.49	0.04	hypo	Zfpm1	0.24	0.03	hypo
Gm20305	0.89	0.06	hypo	Gm45353	0.76	0.37	hypo
Gm23690	0.82	0.36	hypo	Trhr2	0.82	0.28	hypo
Gm17968	0.79	0.20	hypo	Cyba	0.73	0.23	hypo
Gm37214	0.86	0.55	hypo	Gm20735	0.85	0.29	hypo
Eprs	0.23	0.04	hypo	Rnf166	0.25	0.04	hypo
Gm37912	0.36	0.05	hypo	Gm16378	0.84	0.40	hypo
Gm19058	0.43	0.02	hypo	2810013P06Rik	0.13	0.02	hypo
Cenpf	0.21	0.01	hypo	Snord68	0.14	0.01	hypo
Gm37168	0.49	0.10	hypo	Rps12-ps9	0.69	0.33	hypo
Gm2272	0.86	0.36	hypo	Cdk10	0.20	0.00	hypo
Gm20203	0.79	0.28	hypo	Gm45842	0.83	0.29	hypo
1700034H15Rik	0.46	0.14	hypo	AC141881.18	0.00	1.00	hyper
Gm13185	0.71	0.14	hypo	n-R5s104	0.69	0.00	hypo
Gm13176	0.90	0.29	hypo	n-R5s105	0.79	0.09	hypo
Gm13193	0.82	0.46	hypo	n-R5s136	0.51	0.30	hypo
Gm13192	0.98	0.51	hypo	Gm22109	1.00	0.00	hypo
4930551O13Rik	0.76	0.19	hypo	n-R5s141	0.90	0.00	hypo
Gm13216	0.94	0.31	hypo	n-R5s142	0.92	0.06	hypo
Gm13199	0.80	0.31	hypo	Gm23284	0.19	0.02	hypo
A230108P19Rik	0.79	0.27	hypo	n-R5s149	0.23	0.02	hypo
Gm13388	0.81	0.35	hypo	Gm45866	0.66	0.16	hypo
Gm38386	0.85	0.38	hypo	Gm17827	0.88	0.11	hypo
Gm10855	0.79	0.38	hypo	Gm15775	0.72	0.28	hypo
Gm13210	0.91	0.56	hypo	Ttc13	0.24	0.05	hypo
Gm13219	0.87	0.21	hypo	Gm48098	0.96	0.35	hypo
Dreg1	0.82	0.11	hypo	Nkiras1	0.17	0.01	hypo
Gm37866	0.55	0.03	hypo	Gm48860	0.80	0.29	hypo
Atp5c1	0.24	0.02	hypo	Gm48239	0.65	0.03	hypo
Fbh1	0.18	0.05	hypo	Gm48603	0.79	0.32	hypo
Gm37255	0.73	0.00	hypo	Gm48601	0.76	0.17	hypo
Gm37780	0.92	0.37	hypo	Gm48437	0.60	0.00	hypo
Gm37160	0.97	0.53	hypo	Gm45521	0.40	0.05	hypo
Gm13312	1.00	0.35	hypo	Cfap70	0.54	0.10	hypo
Gm13314	0.95	0.47	hypo	Mir6946	0.74	0.10	hypo
Gm13362	0.54	0.02	hypo	Ndst2	0.33	0.07	hypo

Gm13369	0.69	0.30	hypo	AC154573.1	0.97	0.65	hypo
Mir7664	0.78	0.35	hypo	Gm47814	0.79	0.22	hypo
Ndor1	0.88	0.25	hypo	Anxa11os	0.78	0.18	hypo
C8g	0.60	0.25	hypo	Cphx1	0.75	0.24	hypo
Fbxw5	0.39	0.08	hypo	Gm34059	0.92	0.50	hypo
Ajm1	0.61	0.06	hypo	Gm2178	0.95	0.33	hypo
Fcnaos	0.36	0.02	hypo	Gm7591	0.64	0.18	hypo
Lcn9	0.42	0.09	hypo	Gm45645	0.62	0.02	hypo
Card9	0.71	0.24	hypo	Cacna2d3	0.12	0.02	hypo
Mir126b	0.88	0.40	hypo	Mir3076	0.71	0.33	hypo
Snora43	0.68	0.20	hypo	Gm15667	0.92	0.33	hypo
Snora43	0.71	0.21	hypo	Mustn1	0.66	0.14	hypo
Dbhos	0.71	0.18	hypo	Gm35823	0.56	0.05	hypo
Mir7578	0.81	0.16	hypo	Galnt15	0.41	0.06	hypo
Gm13373	0.73	0.07	hypo	AC154846.1	0.60	0.02	hypo
Gm13381	0.74	0.29	hypo	Ncoa4	0.52	0.05	hypo
Spaca9	0.59	0.16	hypo	A630023A22Rik	0.58	0.05	hypo
Ak8	0.49	0.17	hypo	Lrit1	0.66	0.08	hypo
Mir3088	0.97	0.16	hypo	Gm6536	0.85	0.19	hypo
Gm13401	0.57	0.06	hypo	Gm34198	0.62	0.10	hypo
Gm9823	0.71	0.20	hypo	Gm49125	0.80	0.33	hypo
Coq4	0.46	0.05	hypo	Gm10371	0.64	0.18	hypo
Urm1	0.58	0.16	hypo	Gm24378	0.93	0.42	hypo
Mir219a-2	0.36	0.13	hypo	Gm49190	0.73	0.04	hypo
Mir219b	0.59	0.27	hypo	Gm49004	0.62	0.19	hypo
Mir6997	0.80	0.26	hypo	Gm6616	0.01	0.18	hyper
Zdhhc12	0.17	0.00	hypo	Gm41148	0.81	0.10	hypo
Gm28038	0.70	0.18	hypo	Zfp219	0.60	0.05	hypo
Fnbp1	0.19	0.02	hypo	Traj44	0.43	0.00	hypo
Gm16534	0.38	0.07	hypo	Gm43304	0.91	0.43	hypo
Mir7674	0.73	0.25	hypo	Gm17606	0.55	0.11	hypo
Pip5kl1	0.85	0.26	hypo	Mir6948	0.87	0.50	hypo
Mir8093	0.80	0.32	hypo	4930579G18Rik	0.76	0.25	hypo
6330409D20Rik	0.64	0.28	hypo	Homez	0.82	0.23	hypo
Ttc16	0.30	0.07	hypo	Fitm1	0.63	0.11	hypo
Lrsam1	0.34	0.07	hypo	Irf9	0.84	0.21	hypo
Rpl12	0.51	0.11	hypo	Ipo4	0.72	0.27	hypo
Snora65	0.24	0.05	hypo	Gm49378	0.79	0.28	hypo
Garnl3	0.57	0.06	hypo	Gm49299	0.46	0.04	hypo
Gm13408	0.95	0.23	hypo	Gmpr2	0.42	0.03	hypo
AL929106.1	0.73	0.22	hypo	Gm8983	0.54	0.26	hypo
Gm24905	0.25	0.64	hyper	Gm29717	0.70	0.03	hypo

Gm13445	0.50	0.13	hypo	Ift88os	0.78	0.15	hypo
Dab2ip	0.42	0.04	hypo	Gm20430	0.49	0.11	hypo
Gm10829	0.48	0.09	hypo	Gm47694	0.92	0.17	hypo
Rabgap1	0.37	0.01	hypo	Gucy1b2	0.44	0.03	hypo
Gm13584	0.68	0.20	hypo	Gm49390	0.61	0.12	hypo
Gm13525	0.83	0.08	hypo	Gm9086	0.83	0.13	hypo
Gm13480	0.52	0.07	hypo	Gm10032	0.50	0.06	hypo
Gm13548	0.65	0.00	hypo	Kctd9	0.24	0.03	hypo
Wdsub1	0.63	0.08	hypo	Mir6539	0.92	0.50	hypo
Gm13574	0.72	0.06	hypo	Gm31227	0.90	0.28	hypo
4933409G03Rik	0.84	0.25	hypo	Ccar2	0.78	0.12	hypo
Gm37651	0.75	0.16	hypo	Gm49417	0.76	0.20	hypo
Rapgef4os2	0.92	0.17	hypo	Gm22725	0.75	0.22	hypo
Hspe1-ps5	0.87	0.33	hypo	Nudt18	0.40	0.09	hypo
Gm13666	0.89	0.29	hypo	Dok2	0.38	0.03	hypo
AL928867.1	0.91	0.38	hypo	Lrch1	0.74	0.24	hypo
Gm13938	0.90	0.36	hypo	Gm18147	0.54	0.08	hypo
Gm13944	0.36	0.02	hypo	Snora31	0.45	0.06	hypo
Gm25420	0.72	0.07	hypo	Gm49011	0.87	0.04	hypo
Selenoh	0.57	0.07	hypo	Gm30970	0.94	0.26	hypo
Tmx2	0.78	0.11	hypo	Gm49042	0.54	0.14	hypo
Gm13716	0.69	0.33	hypo	Mir759	0.63	0.10	hypo
Gm13772	0.84	0.35	hypo	Gm49199	0.81	0.17	hypo
Arfgap2	0.76	0.29	hypo	Gm49220	0.84	0.22	hypo
Mdk	0.59	0.13	hypo	AC154477.1	0.65	0.30	hypo
Mir6999	0.83	0.20	hypo	Slain1os	0.74	0.12	hypo
Gm9821	0.78	0.34	hypo	Ednrb	0.64	0.17	hypo
Gm13919	0.62	0.16	hypo	Gm16259	0.90	0.33	hypo
Gm13920	0.75	0.18	hypo	5430440P10Rik	0.58	0.12	hypo
Mir1902	0.86	0.09	hypo	Mir18	0.28	0.01	hypo
Gm29053	0.86	0.20	hypo	Mir19a	0.39	0.01	hypo
Gm14207	0.16	0.01	hypo	Mir20a	0.56	0.06	hypo
Nusap1	0.59	0.10	hypo	Mir92-1	0.81	0.26	hypo
Mir7665	0.79	0.37	hypo	1700044C05Rik	0.51	0.06	hypo
Pla2g4b	0.78	0.35	hypo	Gm49033	0.78	0.23	hypo
Lrrc57	0.32	0.05	hypo	Gm23302	0.87	0.40	hypo
Haus2	0.28	0.01	hypo	Mir6391	0.71	0.24	hypo
4930583P06Rik	1.00	0.20	hypo	Gm49768	0.92	0.27	hypo
Slc12a1	0.48	0.06	hypo	1810041H14Rik	0.42	0.08	hypo
Zfp661	0.12	0.01	hypo	Gm6254	0.86	0.28	hypo
Bcl2l11	0.31	0.06	hypo	Gm5089	0.57	0.21	hypo
Gm4430	0.46	0.09	hypo	Gm19212	0.65	0.02	hypo

Gm14023	0.19	0.02	hypo	Mmp7	0.74	0.03	hypo
Gm23187	0.74	0.35	hypo	Tmem123	0.28	0.00	hypo
Gm23650	0.73	0.29	hypo	Gm20416	0.73	0.04	hypo
Snord57	0.78	0.29	hypo	Gm22815	0.83	0.23	hypo
Vps16	0.17	0.03	hypo	Gm10706	0.49	0.12	hypo
4930473A02Rik	0.40	0.03	hypo	Gm16302	0.41	0.14	hypo
Avp	0.59	0.07	hypo	Gm23455	0.80	0.28	hypo
Rpl23a-ps4	1.00	0.41	hypo	Gm24067	0.21	0.01	hypo
Gm14104	0.37	0.04	hypo	Gm50491	0.69	0.10	hypo
Gm14209	0.74	0.19	hypo	Mir7081	0.81	0.33	hypo
Gm14061	0.60	0.03	hypo	Mir199a-1	0.79	0.32	hypo
Dzank1	0.13	0.01	hypo	Mir7083	0.86	0.38	hypo
Cst7	0.78	0.25	hypo	Gm49318	0.32	0.05	hypo
Gm14142	0.88	0.39	hypo	Zfp653	0.40	0.06	hypo
Gm14199	0.68	0.26	hypo	Elof1	0.33	0.09	hypo
Dnmt3bos	0.70	0.28	hypo	Gm48478	0.78	0.12	hypo
Bpifb3	0.51	0.15	hypo	Zfp810	0.21	0.00	hypo
Gm22334	0.88	0.31	hypo	Gm18225	0.91	0.44	hypo
Gm28036	0.79	0.23	hypo	Gm48646	0.92	0.45	hypo
Rpl21-ps9	0.85	0.25	hypo	Gm16165	0.86	0.07	hypo
Gm14168	0.50	0.11	hypo	Gm16164	0.90	0.31	hypo
5430405H02Rik	0.14	0.02	hypo	Gm8031	0.96	0.26	hypo
Gm14277	0.83	0.30	hypo	Gm25861	0.91	0.23	hypo
Src	0.39	0.04	hypo	Gm47436	0.68	0.06	hypo
Gm14286	0.79	0.24	hypo	2610105M22Rik	0.76	0.25	hypo
Nnat	0.60	0.19	hypo	Gm47229	0.82	0.28	hypo
Gm24411	0.90	0.42	hypo	Gm48293	0.98	0.37	hypo
Gm26003	0.81	0.36	hypo	AC158355.2	0.33	0.06	hypo
Adig	0.68	0.04	hypo	AC158355.1	0.69	0.14	hypo
Mir3474	0.60	0.18	hypo	Tbcel	0.65	0.09	hypo
0610039K10Rik	0.40	0.05	hypo	Gm30015	0.48	0.01	hypo
Mir7678	0.33	0.05	hypo	C1qtnf5	0.76	0.28	hypo
Gm14302	0.15	0.01	hypo	Mir7085	0.83	0.30	hypo
Wfdc11	0.47	0.03	hypo	Gm47231	0.33	0.05	hypo
Gm11468	0.50	0.11	hypo	Gm47239	0.60	0.23	hypo
Gm14268	0.74	0.26	hypo	2610028D06Rik	0.73	0.28	hypo
Gm14267	0.81	0.33	hypo	Gm16536	0.81	0.49	hypo
Gm14291	0.59	0.09	hypo	Tagln	0.66	0.22	hypo
Gm11473	0.97	0.67	hypo	A830035O19Rik	0.64	0.11	hypo
Tmem189	0.21	0.06	hypo	Pafah1b2	0.24	0.05	hypo
Mocs3	0.15	0.01	hypo	Gm47544	0.14	0.00	hypo
Gm9873	0.92	0.43	hypo	Gm47197	0.79	0.19	hypo

Bcas1os1	0.62	0.14	hypo	A730065G17Rik	0.60	0.15	hypo
Cyp24a1	0.19	0.01	hypo	Il18	0.89	0.05	hypo
4930470P17Rik	0.51	0.02	hypo	Ppp2r1b	0.87	0.38	hypo
Gm14264	0.78	0.25	hypo	Gm16124	0.47	0.07	hypo
Gm14455	0.14	0.01	hypo	Gm47887	0.63	0.13	hypo
Fam209	0.67	0.20	hypo	D9Wsu149	0.77	0.08	hypo
Gm20490	0.56	0.09	hypo	Mir6385	0.72	0.16	hypo
Gm14453	0.84	0.24	hypo	Stoml1	0.67	0.00	hypo
Mir6340	0.81	0.13	hypo	Gm49759	0.81	0.27	hypo
Gm14300	0.73	0.29	hypo	Gm47676	0.56	0.09	hypo
4921531C22Rik	0.15	0.02	hypo	Gm22099	0.89	0.44	hypo
Adrm1	0.26	0.06	hypo	Larp6	0.48	0.07	hypo
Mir133a-2	0.62	0.09	hypo	Glce	0.22	0.01	hypo
Slco4a1	0.48	0.12	hypo	Mir5133	0.21	0.01	hypo
AL732560.1	0.73	0.08	hypo	Gm47274	0.73	0.09	hypo
Slc17a9	0.75	0.10	hypo	Aagab	0.26	0.05	hypo
Ptk6	0.53	0.10	hypo	Smad3	0.24	0.03	hypo
Uckl1os	0.88	0.06	hypo	Gm18541	0.89	0.28	hypo
Flicr	0.62	0.25	hypo	AC160118.1	0.54	0.07	hypo
Foxp3	0.62	0.25	hypo	Gm48855	0.72	0.11	hypo
Praf2	0.77	0.22	hypo	Gm39363	0.76	0.14	hypo
Rbm3os	0.76	0.39	hypo	Mtfmt	0.39	0.00	hypo
Ndufb11	0.47	0.02	hypo	Spg21	0.69	0.21	hypo
Rbm10	0.63	0.17	hypo	Ciao2a	0.28	0.04	hypo
Gm14558	1.00	0.35	hypo	Fbxl22	0.79	0.20	hypo
Gm6023	0.68	0.21	hypo	Tln2	0.93	0.32	hypo
Zfp36l1-ps	0.84	0.32	hypo	Gm15511	0.70	0.25	hypo
Cul4b	0.89	0.06	hypo	Gm37660	0.85	0.12	hypo
Gm14662	0.23	0.00	hypo	Gm37955	0.79	0.16	hypo
Gm8401	0.82	0.06	hypo	AC158997.1	0.60	0.13	hypo
Ssr4	0.22	0.00	hypo	Gm37611	0.90	0.25	hypo
Gm14808	0.46	0.09	hypo	Wdr72	0.58	0.06	hypo
Otud6a	1.00	0.20	hypo	mmu-mir-1970c	0.68	0.13	hypo
8030474K03Rik	0.39	0.12	hypo	Gm26903	0.43	0.00	hypo
Gm7405	0.78	0.00	hypo	5730403I07Rik	0.59	0.00	hypo
Trpc5	0.02	0.28	hyper	Gm47818	0.59	0.12	hypo
Gm6568	0.90	0.26	hypo	Gm47824	0.70	0.00	hypo
G530011O06Rik	0.57	0.25	hypo	Gm26377	0.40	0.05	hypo
Asmt	0.94	0.69	hypo	Bckdhb	0.34	0.04	hypo
Lrrc34	0.54	0.03	hypo	Tpbg	0.31	0.00	hypo
Mir7008	0.84	0.29	hypo	Gm10163	0.79	0.37	hypo
Gm37834	0.57	0.12	hypo	Plscr2	0.89	0.28	hypo

Gm37592	0.75	0.13	hypo	Dipk2a	0.16	0.02	hypo
Gm15574	0.76	0.28	hypo	Gm24178	0.65	0.19	hypo
Gm43080	0.72	0.17	hypo	Gm50488	0.62	0.11	hypo
B230207O21Rik	0.71	0.22	hypo	Gm19325	0.69	0.13	hypo
Gm43079	0.84	0.34	hypo	Gm28530	0.56	0.10	hypo
Rps23-ps1	0.58	0.12	hypo	4930579K19Rik	0.27	0.03	hypo
Gm44524	0.91	0.35	hypo	Gm37917	0.91	0.14	hypo
Jade1	0.38	0.00	hypo	Gm8641	0.47	0.04	hypo
2610316D01Rik	0.17	0.02	hypo	Gm28166	0.44	0.08	hypo
Gm10729	0.67	0.09	hypo	Gm29143	0.79	0.40	hypo
Gm38034	0.71	0.08	hypo	Gm47468	0.44	0.05	hypo
AC121850.1	0.86	0.27	hypo	Gm37166	0.57	0.15	hypo
Gm16206	0.33	0.08	hypo	Gm16252	0.66	0.17	hypo
Ccdc169	0.81	0.17	hypo	Gm32743	0.76	0.30	hypo
Gm43549	1.00	0.00	hypo	Gm28305	0.39	0.00	hypo
Gpr171	0.87	0.24	hypo	Acad11	0.20	0.04	hypo
P2ry13	0.91	0.10	hypo	Hmgb1-rs16	0.78	0.24	hypo
P2ry12	0.86	0.23	hypo	Gm38077	0.77	0.16	hypo
Gm16129	0.97	0.32	hypo	Nek11	0.44	0.10	hypo
Gm35106	0.76	0.26	hypo	Pik3r4	0.65	0.07	hypo
Gm10723	0.83	0.18	hypo	Mirlet7g	0.74	0.11	hypo
Gm17213	0.87	0.41	hypo	Gm29123	0.82	0.42	hypo
Gm3513	0.87	0.45	hypo	Zmynd10	0.47	0.16	hypo
Gm37973	0.88	0.35	hypo	Gm9917	0.22	0.02	hypo
Gm43839	0.94	0.57	hypo	Lsmem2	0.65	0.30	hypo
Gm10710	0.24	0.03	hypo	Gm38150	0.76	0.15	hypo
Tmem154	0.28	0.02	hypo	Gm19721	0.32	0.08	hypo
Gm37726	1.00	0.13	hypo	Uba7	0.69	0.16	hypo
Sh3d19	0.33	0.05	hypo	4921523L03Rik	0.41	0.07	hypo
Prss48	0.64	0.24	hypo	Gm20662	0.48	0.10	hypo
Gm25188	0.96	0.17	hypo	Rnf123	0.83	0.30	hypo
Snord73b	0.21	0.02	hypo	Amigo3	0.71	0.28	hypo
Gm37933	0.20	0.02	hypo	Gm37568	0.88	0.40	hypo
Gm37876	0.93	0.35	hypo	Gm37963	0.49	0.09	hypo
Lrrc71	0.54	0.22	hypo	Usp19	0.29	0.08	hypo
Naxe	0.56	0.18	hypo	Gm24259	0.88	0.29	hypo
Pmf1	0.80	0.19	hypo	Gm37284	0.80	0.25	hypo
Lmna	0.43	0.13	hypo	Mir711	0.60	0.16	hypo
Mir1905	0.37	0.11	hypo	Nme6	0.71	0.08	hypo
Rab25	0.67	0.11	hypo	Gm43622	0.48	0.15	hypo
Rusc1	0.62	0.13	hypo	5830462I19Rik	0.85	0.33	hypo
Gm22935	0.75	0.41	hypo	Ngp	0.66	0.14	hypo

Scamp3	0.74	0.12	hypo	Myl3	0.30	0.02	hypo
Gm16069	0.40	0.04	hypo	Prss46	0.66	0.13	hypo
Fam189b	0.64	0.12	hypo	Mlh1	0.16	0.01	hypo
Gm43737	0.72	0.31	hypo	Gm47950	0.84	0.24	hypo
Pmvk	0.76	0.20	hypo	Gm4665	0.90	0.28	hypo
Mir7012	0.91	0.42	hypo	Stmn1-rs1	0.87	0.31	hypo
Slc27a3	0.57	0.16	hypo	Gm18935	0.81	0.16	hypo
Gm18296	0.51	0.00	hypo	Gm45897	0.79	0.34	hypo
Selenbp1	0.62	0.12	hypo	Mir26a-1	0.77	0.26	hypo
Gm24920	0.95	0.09	hypo	Plcd1	0.35	0.08	hypo
Gm16740	0.64	0.20	hypo	Acaa1a	0.24	0.01	hypo
Mllt11	0.76	0.31	hypo	Gm47050	0.55	0.13	hypo
Bnipl	0.67	0.05	hypo	Gm47068	0.78	0.28	hypo
Gm37500	0.59	0.00	hypo	Gm47092	0.87	0.33	hypo
Ecm1	0.70	0.24	hypo	Higd1a	0.59	0.00	hypo
Mir7014	0.83	0.23	hypo	Gask1a	0.57	0.09	hypo
Tars2	0.91	0.14	hypo	Topaz1	0.85	0.37	hypo
Gm43375	0.78	0.33	hypo	Gm9856	0.58	0.12	hypo
Gm17690	0.48	0.07	hypo	Exosc7	0.23	0.05	hypo
Gm22027	0.81	0.12	hypo	Gm23323	0.43	0.05	hypo
AC093350.1	0.26	0.04	hypo	Sfi1	0.45	0.31	hypo
Gm43534	0.51	0.16	hypo	Gm12735	0.40	0.24	hypo
Itga10	0.76	0.30	hypo	Gm11944	0.78	0.28	hypo
Mir7225	0.66	0.28	hypo	Selenom	0.76	0.30	hypo
Gm42819	0.89	0.13	hypo	Smtn	0.21	0.00	hypo
Wdr3	0.31	0.05	hypo	Gm24013	0.73	0.21	hypo
Gm42938	0.80	0.16	hypo	Gm11955	0.67	0.21	hypo
Gm42937	0.82	0.25	hypo	Castor1	0.35	0.06	hypo
Casq2	0.41	0.00	hypo	Gm11956	0.46	0.04	hypo
Gm19202	0.67	0.23	hypo	Gm11962	0.68	0.29	hypo
Gm43149	0.78	0.06	hypo	Mrps24	0.63	0.20	hypo
Dclre1b	0.25	0.02	hypo	Pgam2	0.91	0.36	hypo
Wnt2b	0.37	0.02	hypo	Gm11967	0.69	0.28	hypo
Gm9515	0.64	0.04	hypo	Npc1l1	0.64	0.03	hypo
Gstm7	0.58	0.19	hypo	Mir7651	0.78	0.34	hypo
Cyb561d1	0.88	0.29	hypo	Gm24313	0.88	0.45	hypo
Gm12523	0.59	0.13	hypo	Gm11978	0.62	0.29	hypo
Gm43099	0.60	0.14	hypo	Ccdc201	0.13	0.47	hyper
Gm12525	0.79	0.31	hypo	Gm25058	0.79	0.29	hypo
Gm12522	0.22	0.02	hypo	Wdr92	1.00	0.32	hypo
Gm43106	0.89	0.18	hypo	Gm12036	0.81	0.11	hypo
Gm6649	0.91	0.35	hypo	9130230N09Rik	0.26	0.05	hypo

Gm31651	0.69	0.00	hypo	Pnpt1	0.51	0.00	hypo
Gm29736	0.84	0.32	hypo	Gm12094	0.93	0.13	hypo
Gm42927	0.82	0.14	hypo	Gm22588	0.85	0.23	hypo
Gm42777	0.90	0.08	hypo	Gm12119	0.92	0.05	hypo
Gm42544	0.65	0.21	hypo	Gm12124	0.60	0.09	hypo
Gm43729	0.91	0.05	hypo	Gm12146	0.96	0.32	hypo
6330410L21Rik	0.54	0.14	hypo	Fabp6	0.82	0.14	hypo
Rrh	0.91	0.39	hypo	Gm12149	0.83	0.14	hypo
Gm43210	0.88	0.18	hypo	Gm12568	0.79	0.33	hypo
Etnppl	0.84	0.17	hypo	Snord95	0.61	0.07	hypo
Gm43522	0.35	0.05	hypo	Maml1	0.42	0.09	hypo
5830437K03Rik	0.02	0.18	hyper	Gm12196	0.76	0.17	hypo
Gm43430	0.85	0.32	hypo	Gm12205	0.87	0.11	hypo
Gm43446	0.77	0.17	hypo	Mir3061	0.77	0.11	hypo
Col24a1	0.75	0.11	hypo	A630014C17Rik	0.56	0.19	hypo
Gm43408	0.56	0.03	hypo	Gm12208	0.81	0.27	hypo
Rpl36a-ps2	0.47	0.09	hypo	Gm17334	0.77	0.26	hypo
Gm31881	0.54	0.17	hypo	Gm12221	0.86	0.34	hypo
Gm43185	0.98	0.46	hypo	Acsl6	0.68	0.08	hypo
Miga1	0.42	0.03	hypo	Gm12226	0.84	0.34	hypo
Gm42967	0.83	0.04	hypo	Gm12225	1.00	0.23	hypo
St6galnac5	0.65	0.04	hypo	Anxa6	0.47	0.02	hypo
4930597L12Rik	0.28	0.01	hypo	Mir7652	0.72	0.16	hypo
Asb17os	0.76	0.18	hypo	Gm12236	0.73	0.28	hypo
Snord45b	0.62	0.09	hypo	Gm12246	0.79	0.31	hypo
Gm42540	0.86	0.20	hypo	Trim17	0.13	0.00	hypo
Mir186	0.81	0.16	hypo	H2aw	0.13	0.00	hypo
Gm11832	0.89	0.40	hypo	Trim11	0.20	0.01	hypo
Gm12364	0.60	0.14	hypo	Gm10435	0.68	0.28	hypo
Gm12381	0.88	0.57	hypo	Gjc2	0.67	0.11	hypo
Gm12390	0.96	0.07	hypo	Mrpl55	0.68	0.13	hypo
Gm12401	0.97	0.22	hypo	Gm12714	0.50	0.12	hypo
Enho	0.56	0.07	hypo	Med9os	0.25	0.05	hypo
Rpp25l	0.19	0.02	hypo	Mir6921	0.68	0.27	hypo
Dctn3	0.55	0.20	hypo	Gm12265	1.00	0.37	hypo
Sigmar1	0.83	0.30	hypo	Gm12267	0.98	0.48	hypo
Car9	0.64	0.23	hypo	Top3a	0.34	0.07	hypo
Tpm2	0.32	0.03	hypo	Mapk7	0.28	0.00	hypo
Gm12677	0.76	0.24	hypo	Pigl	0.24	0.07	hypo
Frmpd1os	0.65	0.23	hypo	Gm12289	0.91	0.25	hypo
Dcaf10	0.18	0.02	hypo	Hs3st3a1	0.80	0.14	hypo
Gm12408	0.73	0.20	hypo	Gm12294	0.91	0.57	hypo

Ccdc180	0.45	0.04	hypo	Gm12297	0.79	0.11	hypo
Gm12506	0.68	0.23	hypo	2310065F04Rik	0.61	0.00	hypo
2310081O03Rik	0.91	0.24	hypo	C78197	0.60	0.23	hypo
Gm25053	0.79	0.26	hypo	Rpl26	0.17	0.00	hypo
Frrs1l	0.32	0.02	hypo	Mir3062	0.66	0.18	hypo
Gm11483	0.82	0.35	hypo	Gm22442	0.77	0.37	hypo
AL691484.1	0.80	0.36	hypo	Kdm6bos	0.62	0.39	hypo
Gm26566	0.46	0.10	hypo	Dnah2os	0.83	0.36	hypo
Gm10581	0.22	0.03	hypo	Efnb3	0.67	0.32	hypo
Gm13290	0.21	0.03	hypo	Mir1934	0.73	0.30	hypo
4930456L15Rik	0.65	0.14	hypo	Cd68	0.80	0.23	hypo
Gm12715	0.06	0.55	hyper	Gabarap	0.82	0.36	hypo
Gm17662	0.83	0.08	hypo	Dvl2	0.43	0.17	hypo
Gm12721	0.95	0.16	hypo	Mir324	0.70	0.32	hypo
Usp24	0.26	0.02	hypo	Mir7115	0.86	0.19	hypo
Gm12744	0.55	0.04	hypo	Cxcl16	0.69	0.33	hypo
Gm12786	0.92	0.26	hypo	Zmynd15	0.65	0.12	hypo
Gm12870	0.93	0.40	hypo	4930544D05Rik	0.43	0.03	hypo
Gm12869	0.70	0.26	hypo	Rnf167	0.26	0.05	hypo
Gm20731	0.69	0.25	hypo	Mettl16	0.41	0.06	hypo
8030443G20Rik	0.73	0.18	hypo	Sgsm2	0.42	0.10	hypo
Gm28864	0.85	0.14	hypo	Tsr1	0.19	0.05	hypo
Gm29434	0.80	0.40	hypo	Gm12333	0.76	0.10	hypo
Lurap1	0.71	0.11	hypo	Gm12340	0.95	0.16	hypo
1700021J08Rik	0.77	0.17	hypo	Slc6a4	0.17	0.00	hypo
Gm22335	0.95	0.18	hypo	Gm12345	0.75	0.20	hypo
Gm23382	0.81	0.33	hypo	mmu-mir-12181	0.25	0.04	hypo
Mir7226	0.79	0.46	hypo	Tlcd1	0.32	0.03	hypo
Tie1	0.73	0.07	hypo	Snord42a	0.44	0.00	hypo
Mir1957a	0.83	0.20	hypo	Snord4a	0.24	0.00	hypo
Gm12867	0.78	0.22	hypo	Rskr	0.90	0.40	hypo
Gm12955	0.73	0.19	hypo	Gm11196	0.98	0.08	hypo
Gm12957	0.79	0.34	hypo	Rab11fip4os1	0.95	0.40	hypo
AL645563.1	0.90	0.44	hypo	Rab11fip4os2	0.74	0.23	hypo
Guca2b	0.74	0.10	hypo	Mir365-2	0.75	0.32	hypo
Edn2	0.68	0.08	hypo	Gm11203	0.97	0.29	hypo
Gm25788	0.84	0.31	hypo	Ccl1	0.41	0.00	hypo
Gm12924	0.79	0.31	hypo	Rffl	0.57	0.06	hypo
Gm12903	0.90	0.17	hypo	Nle1	0.48	0.05	hypo
Gm12917	0.82	0.18	hypo	Unc45b	0.61	0.00	hypo
Cdca8	0.75	0.20	hypo	Rasl10b	0.79	0.24	hypo
Gm12930	0.30	0.07	hypo	Ggnbp2	0.40	0.04	hypo

Oscp1	0.57	0.28	hypo	Akirin1-ps	0.86	0.19	hypo
Trappc3	0.21	0.00	hypo	Bcas3os1	0.90	0.24	hypo
Adprs	0.14	0.01	hypo	Gm11443	0.86	0.33	hypo
Gm12936	0.90	0.40	hypo	1110028F11Rik	0.61	0.11	hypo
Gm12944	0.94	0.34	hypo	Acsf2	0.57	0.12	hypo
Gjb4	0.38	0.05	hypo	Gm11540	0.91	0.11	hypo
A3galt2	0.58	0.06	hypo	Hoxb4	0.30	0.01	hypo
Fndc5	0.31	0.00	hypo	D030028A08Rik	0.31	0.07	hypo
Fam229a	0.29	0.02	hypo	Gm11533	0.84	0.25	hypo
Gm12966	0.38	0.06	hypo	Osbpl7	0.87	0.29	hypo
Pef1	0.57	0.00	hypo	Gm11583	0.69	0.16	hypo
Gm12971	0.69	0.06	hypo	Gm11594	0.77	0.24	hypo
Gm12970	0.54	0.04	hypo	Mir6927	0.70	0.18	hypo
Gm50475	0.22	0.08	hypo	Gm11633	0.42	0.02	hypo
Rps15a-ps4	0.71	0.24	hypo	Gm11632	0.79	0.23	hypo
Gm12999	0.84	0.14	hypo	Gm12352	0.89	0.29	hypo
Gm22767	0.91	0.21	hypo	Csf3	0.52	0.13	hypo
Cd164l2	0.60	0.19	hypo	Gm22059	0.76	0.35	hypo
Gm12977	0.68	0.24	hypo	Nr1d1	0.53	0.09	hypo
Lin28a	0.38	0.06	hypo	Krt20	0.66	0.16	hypo
Zpld2	0.46	0.11	hypo	Mir7116	0.57	0.15	hypo
Gm30191	0.15	0.01	hypo	Dhx58	0.83	0.37	hypo
Mir6403	0.79	0.34	hypo	Hsd17b1	0.73	0.20	hypo
Gm16224	0.34	0.08	hypo	Wnk4	0.44	0.13	hypo
Gm12991	0.73	0.13	hypo	Gm11626	0.59	0.20	hypo
Gm12990	0.70	0.19	hypo	Rnd2	0.37	0.01	hypo
Mir700	0.85	0.45	hypo	Gm11628	0.87	0.29	hypo
Gm15979	0.62	0.14	hypo	Meioc	0.43	0.14	hypo
Ephb2	0.22	0.02	hypo	Mir6931	0.68	0.19	hypo
Rap1gap	0.76	0.29	hypo	Gm11622	0.89	0.44	hypo
Mir6399	0.82	0.30	hypo	Tlk2	0.82	0.17	hypo
Gm25280	0.32	0.00	hypo	Kcnh6	0.44	0.03	hypo
Gm26226	0.70	0.08	hypo	Gm11646	0.81	0.21	hypo
Gm13031	0.51	0.05	hypo	Limd2	0.77	0.39	hypo
Sdhb	0.59	0.08	hypo	Gm23645	0.95	0.25	hypo
Necap2	0.32	0.03	hypo	Prr29	0.35	0.00	hypo
Gm13047	0.71	0.09	hypo	Milr1	0.90	0.40	hypo
4921514A10Rik	0.73	0.26	hypo	Mir3064	0.71	0.06	hypo
Gm13062	0.68	0.19	hypo	Gm11713	0.72	0.26	hypo
Gm13228	0.70	0.31	hypo	Gm11649	0.47	0.00	hypo
Gm13034	0.03	0.18	hyper	Gm11657	0.95	0.40	hypo
Gm13141	0.64	0.18	hypo	Gm11670	0.64	0.33	hypo

Gm13136	0.04	0.18	hyper	Prkar1a	0.62	0.08	hypo
Gm13054	0.84	0.42	hypo	Map2k6	0.35	0.03	hypo
Gm13207	0.77	0.33	hypo	4732490B19Rik	0.94	0.25	hypo
Gm24002	0.74	0.32	hypo	Gm11691	0.57	0.09	hypo
Exosc10	0.51	0.06	hypo	Btbd17	0.76	0.23	hypo
Gm13203	0.59	0.19	hypo	Mir3968	0.68	0.25	hypo
Gm17029	0.73	0.38	hypo	Gm11695	0.36	0.01	hypo
Gm9506	0.97	0.49	hypo	Mir5621	0.85	0.36	hypo
Cort	0.82	0.33	hypo	Llgl2	0.24	0.02	hypo
Ube4bos2	0.93	0.46	hypo	Smim6	0.66	0.18	hypo
Lzic	0.34	0.03	hypo	Recql5os1	0.77	0.15	hypo
Mir5616	0.72	0.15	hypo	Unc13d	0.68	0.22	hypo
Mir7023	0.71	0.15	hypo	Trim65	0.72	0.20	hypo
Gm13073	0.64	0.16	hypo	Ten1	0.13	0.01	hypo
Gm13091	0.67	0.09	hypo	Cdk3	0.66	0.03	hypo
Uts2	0.82	0.32	hypo	Prpsap1	0.69	0.22	hypo
Tnfrsf25	0.80	0.28	hypo	St6galnac2	0.82	0.41	hypo
Gm13096	0.87	0.13	hypo	Gm16045	0.61	0.04	hypo
Hes3	0.35	0.05	hypo	Afmid	0.24	0.01	hypo
Gm16333	0.63	0.12	hypo	Gm11725	0.76	0.40	hypo
Trp73os	0.89	0.38	hypo	Mir6933	0.85	0.50	hypo
Gm13132	0.77	0.23	hypo	Gm11738	0.55	0.15	hypo
B230104I21Rik	0.39	0.12	hypo	Cep295nl	0.54	0.08	hypo
Gm13133	0.48	0.12	hypo	Engase	0.29	0.03	hypo
Gm10564	0.80	0.33	hypo	Mir1932	0.24	0.02	hypo
Gm10563	0.71	0.15	hypo	Gm11770	0.62	0.25	hypo
Tmem240	0.50	0.07	hypo	Mir6935	0.17	0.03	hypo
Aurkaip1	0.90	0.04	hypo	0610009L18Rik	0.22	0.04	hypo
Mir7658	0.89	0.51	hypo	Mrpl12	0.47	0.08	hypo
Samd11	0.30	0.00	hypo	Anapc11	0.13	0.01	hypo
Rbm48	0.39	0.09	hypo	Gps1	0.21	0.03	hypo
Gm43679	0.84	0.14	hypo	Gm11773	0.75	0.25	hypo
Gm15459	0.88	0.49	hypo	Gm28192	0.76	0.28	hypo
Gm21759	0.92	0.29	hypo	Wdr45b	0.21	0.02	hypo
Gm42490	0.77	0.19	hypo	Gm40655	0.73	0.18	hypo
Gm15610	0.88	0.40	hypo	Gm47813	0.96	0.21	hypo
Gm43489	0.87	0.15	hypo	Gm47549	0.94	0.49	hypo
Gm21009	0.89	0.41	hypo	Gm48375	0.90	0.36	hypo
Lrrc17	0.87	0.17	hypo	Gm48650	0.72	0.17	hypo
Gm15589	0.91	0.53	hypo	Gm48888	0.95	0.37	hypo
Gm10062	0.71	0.06	hypo	Gm5928	0.77	0.11	hypo
Gm43502	0.88	0.25	hypo	Gm48445	0.47	0.08	hypo

AC115028.1	0.78	0.12	hypo	Gm47884	0.88	0.29	hypo
Lmbr1	0.34	0.02	hypo	Gm48338	0.70	0.03	hypo
C79130	0.93	0.13	hypo	AK157302	0.68	0.24	hypo
4632411P08Rik	0.31	0.10	hypo	Vmn1r-ps95	0.95	0.56	hypo
Gm42570	0.88	0.28	hypo	Armh2	0.92	0.26	hypo
Gm5552	0.97	0.33	hypo	Acot13	0.18	0.01	hypo
Adgrf3	0.48	0.08	hypo	A330102I10Rik	0.35	0.02	hypo
Gm16288	1.00	0.10	hypo	Tubb2b	0.38	0.12	hypo
Fam166c	0.45	0.04	hypo	Psmg4	0.79	0.19	hypo
Gm43808	0.68	0.29	hypo	Gm48706	0.75	0.21	hypo
5930420M18Rik	0.71	0.17	hypo	Gm49350	0.83	0.28	hypo
Gm9924	0.25	0.10	hypo	Gm47991	0.70	0.24	hypo
Mpv17	0.59	0.08	hypo	Gm48188	0.90	0.27	hypo
Eif2b4	0.19	0.02	hypo	Gm17364	0.90	0.29	hypo
4930566F21Rik	0.46	0.07	hypo	Gm26877	0.27	0.00	hypo
Mir3473e	0.78	0.29	hypo	Gm48569	0.70	0.20	hypo
Gm20671	0.80	0.16	hypo	1700061E18Rik	0.83	0.19	hypo
G630022F23Rik	0.16	0.00	hypo	Gm22157	0.84	0.38	hypo
Poln	0.24	0.00	hypo	Hivep1	0.42	0.11	hypo
Mfsd10	0.70	0.28	hypo	Gm47125	0.76	0.12	hypo
Nop14	0.10	0.00	hypo	Gm15810	0.58	0.14	hypo
Grk4	0.20	0.02	hypo	Gm47728	0.89	0.23	hypo
Mir3097	0.75	0.29	hypo	Gm33630	0.41	0.08	hypo
4930442P19Rik	0.13	0.02	hypo	Gm47460	0.78	0.09	hypo
Gm43860	0.66	0.27	hypo	Gm24620	0.95	0.52	hypo
Trmt44	0.36	0.00	hypo	A930002C04Rik	0.24	0.01	hypo
Sh3tc1	0.35	0.00	hypo	Gm36550	0.84	0.45	hypo
Psapl1	0.76	0.24	hypo	H2af-ps2	0.52	0.11	hypo
Gm40289	0.55	0.13	hypo	Gm33424	0.59	0.00	hypo
Drd5	0.21	0.03	hypo	Nop16	0.19	0.01	hypo
Mir6414	0.68	0.00	hypo	Gm17617	0.63	0.05	hypo
Gm16401	0.69	0.20	hypo	Mir6943	0.87	0.52	hypo
Gm42428	0.85	0.33	hypo	Mir6944	0.75	0.14	hypo
Gm42427	0.85	0.30	hypo	Mir6945	0.72	0.33	hypo
E430021H15Rik	0.79	0.20	hypo	Gm46416	0.73	0.29	hypo
Gm42982	0.78	0.23	hypo	Gm15911	0.87	0.47	hypo
C130083M11Rik	0.59	0.21	hypo	Catsper3	0.89	0.46	hypo
Gm43685	0.79	0.18	hypo	Gm47071	0.61	0.27	hypo
AC139037.1	0.72	0.09	hypo	Gm17878	0.77	0.18	hypo
Tent2-ps1	0.83	0.13	hypo	Gm10782	0.82	0.38	hypo
Tbc1d1	0.88	0.12	hypo	Gm48357	0.84	0.27	hypo
Gm43721	0.92	0.23	hypo	Gkap1	0.60	0.15	hypo

Gm15823	0.82	0.16	hypo	Mir6369	0.93	0.19	hypo
Gm6044	0.89	0.38	hypo	Hnrnpk	0.43	0.02	hypo
1110003F10Rik	0.88	0.25	hypo	Gm40968	0.64	0.20	hypo
Gm43311	0.93	0.19	hypo	Cts3	0.94	0.16	hypo
Gm43552	0.15	0.00	hypo	Gm7240	0.73	0.03	hypo
C030017G13Rik	1.00	0.41	hypo	Gm47602	0.89	0.28	hypo
Gm43790	0.67	0.15	hypo	Gm16133	0.75	0.15	hypo
Gm43793	0.83	0.06	hypo	Gm10775	0.51	0.05	hypo
Gm43794	0.85	0.12	hypo	Gm48424	0.75	0.30	hypo
Gm43343	0.86	0.34	hypo	Tent4a	0.13	0.01	hypo
Gm43795	0.90	0.16	hypo	Gm48676	0.77	0.20	hypo
Slc30a9	0.38	0.04	hypo	Gm36377	0.77	0.06	hypo
Gm42735	0.89	0.28	hypo	Gm6263	0.49	0.06	hypo
Gm10135	0.78	0.30	hypo	AW495222	0.81	0.16	hypo
Gm34411	0.88	0.24	hypo	Gm48350	0.80	0.35	hypo
Gm42734	0.84	0.24	hypo	Pde8b	0.89	0.35	hypo
Usp46	0.47	0.10	hypo	Gm50469	0.78	0.15	hypo
Apc-ps1	0.75	0.14	hypo	Gfm2	0.39	0.02	hypo
Gm15831	0.32	0.01	hypo	Gm41031	0.84	0.20	hypo
Gm43594	1.00	0.24	hypo	Gm10320	0.53	0.00	hypo
Gm43432	0.62	0.23	hypo	5330431K02Rik	0.75	0.27	hypo
Gm22728	0.92	0.21	hypo	Gm16335	0.98	0.48	hypo
Ppef2	0.75	0.16	hypo	Gm2534	0.94	0.26	hypo
Cxcl11	0.45	0.05	hypo	Gm36638	0.70	0.21	hypo
Gm42604	0.90	0.12	hypo	Gm17160	0.80	0.25	hypo
Gm8013	0.59	0.00	hypo	AC159301.1	0.91	0.29	hypo
Gm43272	0.92	0.35	hypo	Gm26943	0.88	0.28	hypo
Gm42934	0.84	0.33	hypo	Gm37907	0.67	0.16	hypo
Gm43787	0.88	0.16	hypo	Gm10734	0.69	0.26	hypo
Gm42933	0.78	0.13	hypo	Parp8	0.17	0.01	hypo
Gm42619	0.73	0.11	hypo	3110070M22Rik	0.70	0.42	hypo
Gm26703	0.55	0.08	hypo	Gm48265	0.55	0.33	hypo
Gm42900	0.70	0.22	hypo	Gm48342	0.74	0.03	hypo
Gm42902	0.19	0.01	hypo	Gm18244	0.95	0.46	hypo
Gm9727	0.94	0.20	hypo	4921501I09Rik	0.72	0.13	hypo
Gm34319	0.33	0.08	hypo	Gm3625	0.18	0.01	hypo
Tmem175	0.12	0.02	hypo	Gm23167	1.00	0.32	hypo
Fgfrl1	0.74	0.19	hypo	Gm6736	0.93	0.39	hypo
Gm16019	0.93	0.31	hypo	Gm48227	0.77	0.00	hypo
Gm43759	0.71	0.08	hypo	Gm29968	0.90	0.13	hypo
Gm43246	0.74	0.19	hypo	Gm48904	0.75	0.19	hypo
F830115B05Rik	0.87	0.19	hypo	Gm18024	0.63	0.12	hypo

Gm42161	0.75	0.24	hypo	Gm19046	0.43	0.11	hypo
Aym1	0.69	0.03	hypo	4930428E07Rik	0.68	0.00	hypo
Wscd2	0.75	0.35	hypo	1700060O08Rik	0.82	0.26	hypo
Gm17122	0.77	0.23	hypo	Mir1892	0.89	0.24	hypo
Usp30	0.91	0.25	hypo	Prorp	0.77	0.24	hypo
Gm42913	0.86	0.43	hypo	Gm38103	0.25	0.01	hypo
Acacb	0.44	0.09	hypo	Gm48835	0.81	0.12	hypo
Gm13784	0.66	0.28	hypo	Gm47491	0.83	0.05	hypo
Mir7027	0.76	0.47	hypo	Gm24449	1.00	0.19	hypo
Gm42789	0.86	0.44	hypo	Mir681	0.80	0.18	hypo
Mir7028	0.82	0.50	hypo	Gm10457	0.53	0.16	hypo
Git2	0.34	0.05	hypo	Dact1	0.13	0.00	hypo
Gm9936	0.52	0.04	hypo	Gm33016	0.66	0.07	hypo
Gm13823	0.98	0.39	hypo	Gm40438	0.68	0.13	hypo
Gm10401	0.71	0.31	hypo	Trmt5	0.26	0.03	hypo
Rpl37rt	0.59	0.30	hypo	Rplp2-ps1	0.72	0.23	hypo
Gm13831	0.92	0.42	hypo	Gm34868	0.65	0.08	hypo
Gm13840	0.64	0.26	hypo	Gm48196	0.86	0.16	hypo
Mir7029	0.72	0.32	hypo	Gm47767	0.80	0.24	hypo
Mir7030	0.89	0.42	hypo	Plekhd1os	0.08	0.29	hyper
Gm14508	0.50	0.06	hypo	Gm49654	0.78	0.34	hypo
Gm14507	0.84	0.24	hypo	Gm26571	0.41	0.21	hypo
Gm43637	0.83	0.40	hypo	Gm8385	0.71	0.29	hypo
Gm15728	0.22	0.03	hypo	Pnma1	0.58	0.02	hypo
Gm37028	0.67	0.20	hypo	Gm48573	0.42	0.02	hypo
Gm26411	0.68	0.19	hypo	Fcf1	0.28	0.00	hypo
AC125061.1	0.63	0.09	hypo	Pgf	0.46	0.08	hypo
Gm43785	0.46	0.00	hypo	Gm47817	0.88	0.31	hypo
Tbx3os1	0.17	0.01	hypo	Gm8557	0.90	0.45	hypo
Tbx3os2	0.49	0.06	hypo	4930473H19Rik	0.78	0.28	hypo
Ddx54	0.29	0.05	hypo	Gm18388	0.93	0.44	hypo
Gm42656	0.56	0.17	hypo	Gm8785	0.98	0.13	hypo
Gm24671	0.75	0.22	hypo	3300002A11Rik	0.80	0.07	hypo
Gm42917	0.87	0.22	hypo	Gm47138	0.80	0.21	hypo
Gm43420	0.72	0.16	hypo	Gm47208	0.89	0.23	hypo
Mir7031	0.95	0.43	hypo	Gm47207	0.85	0.27	hypo
Gm15846	0.78	0.30	hypo	Gm48147	0.83	0.19	hypo
P2rx4	0.37	0.06	hypo	Gm48381	0.86	0.26	hypo
Kdm2b	0.72	0.22	hypo	A630072L19Rik	0.82	0.30	hypo
A930024E05Rik	0.62	0.08	hypo	Gm20069	0.74	0.27	hypo
Gm6444	0.49	0.05	hypo	Gm20604	0.54	0.02	hypo
5830487J09Rik	0.74	0.29	hypo	Gm15523	0.47	0.05	hypo

Gm42907	0.36	0.06	hypo	Ddx24	0.55	0.00	hypo
Mir7032	0.85	0.47	hypo	Gm47260	0.69	0.18	hypo
Gm37939	0.85	0.33	hypo	Gm47267	0.83	0.15	hypo
Gm43661	0.80	0.22	hypo	Gsc	0.32	0.05	hypo
Gm16001	0.50	0.12	hypo	Gm49732	0.79	0.25	hypo
Ogfod2	0.53	0.18	hypo	Gm47650	0.79	0.23	hypo
Arl6ip4	0.73	0.30	hypo	Gm2721	0.89	0.16	hypo
Pitpnm2	0.13	0.00	hypo	Gm46376	0.61	0.18	hypo
Pitpnm2os2	0.87	0.21	hypo	Gm46378	0.57	0.20	hypo
Cdk2ap1	0.81	0.15	hypo	Eml1	0.44	0.02	hypo
Gm37415	0.64	0.04	hypo	Gm47980	0.65	0.30	hypo
Dnah10	0.66	0.10	hypo	Mir493	0.81	0.19	hypo
Gm42838	0.80	0.38	hypo	Mir3544	0.89	0.20	hypo
Gm42633	0.61	0.00	hypo	mmu-mir-3070-1	0.88	0.22	hypo
Dhx37	0.32	0.03	hypo	mmu-mir-3070-2	0.88	0.30	hypo
Gm43475	0.51	0.03	hypo	Mir431	0.92	0.50	hypo
4930553I04Rik	0.51	0.12	hypo	Mir433	0.95	0.51	hypo
Gm42979	0.71	0.16	hypo	Mir127	0.88	0.48	hypo
Sumf2	0.81	0.15	hypo	Mir434	0.87	0.63	hypo
Gm42588	0.71	0.26	hypo	Mir432	0.89	0.64	hypo
Gm42992	0.87	0.31	hypo	Mir341	0.93	0.39	hypo
Gm43019	0.95	0.39	hypo	Mir1188	0.93	0.32	hypo
Gm43186	0.72	0.20	hypo	Mir370	0.89	0.34	hypo
2700029L08Rik	0.92	0.44	hypo	Gm24564	0.85	0.29	hypo
Lat2	0.69	0.09	hypo	B830012L14Rik	0.96	0.32	hypo
Gm43372	0.68	0.27	hypo	AF357428	0.93	0.12	hypo
Gm25492	0.79	0.35	hypo	Mir411	0.75	0.29	hypo
Dnajc30	0.40	0.06	hypo	Mir299a	0.81	0.26	hypo
Mir7033	0.72	0.09	hypo	Mir299b	0.90	0.27	hypo
Mir7034	0.50	0.09	hypo	Mir380	0.85	0.44	hypo
Gm16599	0.83	0.31	hypo	Mir1197	0.88	0.33	hypo
Cldn15	0.66	0.28	hypo	Mir323	0.91	0.32	hypo
Znhit1	0.54	0.12	hypo	Mir758	0.88	0.20	hypo
Mir702	0.84	0.28	hypo	Mir329	0.87	0.16	hypo
Gm7284	0.75	0.23	hypo	Mir543	0.73	0.17	hypo
Srrt	0.57	0.02	hypo	Mir495	0.89	0.34	hypo
Gigyf1	0.84	0.31	hypo	Mir300	0.86	0.39	hypo
Mir6418	0.39	0.10	hypo	Mir381	0.86	0.35	hypo
Gm36266	0.64	0.17	hypo	Mir487b	0.98	0.48	hypo
Sap25	0.61	0.18	hypo	Mir539	0.97	0.60	hypo
Nyap1	0.44	0.12	hypo	Mir382	0.92	0.39	hypo
Mepce	0.37	0.08	hypo	Mir134	0.92	0.35	hypo

Map11	0.68	0.19	hypo	Mir668	0.92	0.35	hypo
Stag3	0.38	0.02	hypo	Mir485	0.91	0.35	hypo
6330403L08Rik	0.28	0.05	hypo	Mir453	0.91	0.35	hypo
AC140216.1	0.80	0.33	hypo	Mir496a	0.82	0.16	hypo
Gm42424	0.92	0.52	hypo	Mir377	0.69	0.21	hypo
Mir7037	0.92	0.40	hypo	Mir409	0.72	0.29	hypo
Gm16120	0.75	0.14	hypo	Mir412	0.73	0.31	hypo
Mir7038	0.75	0.19	hypo	Mir369	0.74	0.33	hypo
Amz1	0.58	0.14	hypo	Mir410	0.69	0.38	hypo
Gm6433	0.78	0.27	hypo	4930511J24Rik	0.52	0.11	hypo
Gm26970	0.93	0.46	hypo	3110009F21Rik	0.70	0.13	hypo
Fbxl18	0.30	0.03	hypo	Gm17111	0.79	0.15	hypo
Olfr718-ps1	0.74	0.00	hypo	Rcor1	0.22	0.05	hypo
0610040B10Rik	0.35	0.05	hypo	Gm48286	0.86	0.21	hypo
Gm20635	0.26	0.03	hypo	Gm15996	0.64	0.20	hypo
Gm42502	0.90	0.41	hypo	Rd3l	0.92	0.24	hypo
Aimp2	0.15	0.00	hypo	Aspg	0.79	0.15	hypo
Gm42421	0.81	0.20	hypo	Kif26a	0.12	0.00	hypo
Rsph10b	0.72	0.22	hypo	Mir6939	0.81	0.21	hypo
Gm15708	0.78	0.08	hypo	Cep170b	0.55	0.12	hypo
AC131728.1	0.51	0.14	hypo	Mir6940	0.79	0.33	hypo
Gm23995	0.72	0.08	hypo	Ahnak2	0.38	0.03	hypo
Mir7039	0.88	0.40	hypo	Mir6941	0.84	0.39	hypo
Gm8494	0.81	0.34	hypo	Crip1	0.64	0.19	hypo
Rps10-ps4	0.87	0.42	hypo	Gm9260	0.52	0.09	hypo
Gm42883	0.89	0.24	hypo	Ncapg2	0.26	0.08	hypo
Gm35648	0.82	0.15	hypo	Gm6171	0.80	0.43	hypo
Gm43151	0.85	0.17	hypo	Gm9614	0.69	0.30	hypo
Gm20005	0.68	0.14	hypo	Gm4823	0.93	0.36	hypo
Gm36378	0.63	0.17	hypo	Snord72	0.15	0.02	hypo
Gm18753	0.71	0.16	hypo	Gm10250	0.90	0.32	hypo
AC154885.1	0.97	0.49	hypo	Gm19227	0.79	0.23	hypo
Gm43598	0.43	0.00	hypo	Gm10389	0.82	0.41	hypo
Gm43807	0.93	0.39	hypo	Gm2573	0.83	0.07	hypo
Gm43298	0.70	0.08	hypo	Gm18714	0.82	0.33	hypo
Tmem168	0.00	0.26	hyper	Gm49127	0.78	0.11	hypo
Lsm8	0.29	0.00	hypo	Gm2862	0.62	0.08	hypo
Eif3s6-ps4	0.93	0.56	hypo	B230362B09Rik	0.66	0.33	hypo
Gm30270	0.32	0.07	hypo	Gm9891	0.73	0.30	hypo
Gm42573	0.63	0.03	hypo	Gm20555	0.64	0.18	hypo
Gm43629	1.00	0.29	hypo	Gm19883	0.51	0.06	hypo
Gm22267	0.61	0.10	hypo	Tas2r119	0.58	0.00	hypo

Gm37321	0.78	0.11	hypo	Gm49091	0.34	0.03	hypo
Lrrc4	0.58	0.10	hypo	Gm18949	0.83	0.21	hypo
Gm37940	0.71	0.33	hypo	Gm16138	0.67	0.11	hypo
Gm13725	0.89	0.20	hypo	Gm28221	0.53	0.09	hypo
Gm13844	0.97	0.34	hypo	Gm8668	0.78	0.15	hypo
Gm13864	0.95	0.13	hypo	4930447A16Rik	0.75	0.32	hypo
1700025N23Rik	0.80	0.18	hypo	Gm49253	0.92	0.42	hypo
Mgam	0.86	0.23	hypo	1700015H07Rik	0.62	0.03	hypo
Trbv21	0.87	0.43	hypo	Gm41325	0.67	0.16	hypo
Trbj1-1	0.84	0.31	hypo	Gm9920	0.62	0.14	hypo
Trbj1-2	0.85	0.30	hypo	9130401M01Rik	0.57	0.04	hypo
Trbj1-3	0.83	0.29	hypo	9930014A18Rik	0.83	0.21	hypo
Trbj1-4	0.70	0.29	hypo	Gm16308	0.88	0.12	hypo
9430018G01Rik	0.92	0.30	hypo	Gm49457	0.90	0.35	hypo
Mir704	0.73	0.16	hypo	Gm3150	0.84	0.33	hypo
Zfp783	0.59	0.12	hypo	Gm53029	0.73	0.24	hypo
Gimap1os	0.31	0.11	hypo	Scrib	0.67	0.11	hypo
Tmem176b	0.43	0.08	hypo	Mir6952	0.76	0.25	hypo
4930563H07Rik	0.80	0.27	hypo	Spatc1	0.47	0.10	hypo
Gm8039	0.93	0.36	hypo	Mir3079	0.58	0.10	hypo
C530044C16Rik	0.87	0.39	hypo	Oplah	0.39	0.09	hypo
G930045G22Rik	0.53	0.00	hypo	Gm35339	0.63	0.12	hypo
Gm44028	0.88	0.40	hypo	Mir6954	0.74	0.31	hypo
Gm44434	0.85	0.20	hypo	Scx	0.77	0.34	hypo
Gm15572	0.80	0.15	hypo	Gm49550	0.90	0.21	hypo
Gm16499	0.34	0.10	hypo	Gpt	0.83	0.38	hypo
Gm16499	0.24	0.02	hypo	Lrrc14	0.38	0.11	hypo
Aqp1	0.62	0.02	hypo	C030006K11Rik	0.36	0.12	hypo
Gm29848	0.81	0.30	hypo	Gm35933	0.27	0.02	hypo
Igkv1-136	0.88	0.59	hypo	AL603843.1	0.96	0.29	hypo
Igkv12-67	0.89	0.52	hypo	1700109K24Rik	0.80	0.20	hypo
AC155842.1	0.93	0.37	hypo	Gm17638	0.91	0.28	hypo
Gm44604	0.86	0.14	hypo	A730060N03Rik	0.36	0.04	hypo
Gm29438	0.69	0.14	hypo	Mpst	0.23	0.03	hypo
4933431G14Rik	0.76	0.14	hypo	Il2rb	0.88	0.34	hypo
Sftpb	0.63	0.20	hypo	Mir6956	0.57	0.19	hypo
Gm45051	0.67	0.04	hypo	Baiap2l2	0.85	0.39	hypo
Gm45053	0.79	0.15	hypo	Gm16059	0.77	0.24	hypo
Gm44202	0.50	0.03	hypo	Mir7213	0.80	0.33	hypo
Gm31861	0.68	0.14	hypo	AC161199.1	0.51	0.04	hypo
Pole4	0.69	0.10	hypo	Gm19141	0.47	0.02	hypo
2310069B03Rik	0.83	0.38	hypo	Mir6957	0.63	0.02	hypo

Gm6312	0.79	0.27	hypo	Mir5113	0.68	0.13	hypo
Mir7040	0.39	0.01	hypo	Gm6612	0.57	0.18	hypo
Htra2	0.54	0.19	hypo	Gm49522	0.93	0.35	hypo
Dqx1	0.66	0.28	hypo	Aco2	0.28	0.00	hypo
5430434F05Rik	0.82	0.33	hypo	Gm49502	0.66	0.21	hypo
B230319C09Rik	0.67	0.26	hypo	Mir33	0.81	0.24	hypo
Gm33024	0.75	0.00	hypo	Tnfrsf13c	0.67	0.26	hypo
Gm5878	0.27	0.02	hypo	Gm49503	0.79	0.34	hypo
Gm28719	0.44	0.03	hypo	Cyb5r3	0.46	0.02	hypo
Rab43	0.35	0.06	hypo	Gm49463	0.21	0.03	hypo
B130021K23Rik	0.82	0.38	hypo	mmu-mir-12186	0.92	0.43	hypo
Sec61a1	0.22	0.02	hypo	Gm4217	0.64	0.07	hypo
Gm45216	0.81	0.30	hypo	Nup50	0.37	0.05	hypo
4930517G19Rik	0.74	0.24	hypo	Mir1249	0.80	0.27	hypo
Gm43923	0.90	0.18	hypo	Wnt7b	0.22	0.01	hypo
Mir7041	0.80	0.38	hypo	Mirlet7b	0.72	0.30	hypo
Gm26175	0.89	0.39	hypo	Ppara	0.38	0.04	hypo
Gm44196	0.70	0.00	hypo	Gm15722	0.87	0.32	hypo
mmu-mir-12201	0.77	0.27	hypo	A930001M01Rik	0.84	0.34	hypo
Gm20705	0.79	0.25	hypo	Creld2	0.22	0.04	hypo
Gm6565	0.87	0.43	hypo	Tubgcp6	0.25	0.04	hypo
Gm44116	0.72	0.11	hypo	Mapk11	0.45	0.13	hypo
9530086O07Rik	0.93	0.25	hypo	Mir7118	0.88	0.46	hypo
Gm43947	0.90	0.19	hypo	Mir6958	0.91	0.38	hypo
Gm23339	1.00	0.08	hypo	Mir6959	0.91	0.37	hypo
Cpne9	0.61	0.17	hypo	Cpt1b	0.81	0.30	hypo
Gm44280	0.79	0.33	hypo	Gm3861	0.49	0.07	hypo
Prrt3	0.41	0.05	hypo	Yaf2	0.14	0.02	hypo
AC149276.1	0.85	0.24	hypo	Gm23129	0.92	0.15	hypo
Gm4640	0.91	0.37	hypo	Ano6	0.31	0.04	hypo
4933440N22Rik	0.16	0.03	hypo	Pced1b	0.25	0.08	hypo
Gm23908	1.00	0.30	hypo	Gm49443	0.92	0.45	hypo
Gm20692	0.66	0.08	hypo	Mir1291	0.63	0.06	hypo
Gm43916	0.73	0.12	hypo	Adcy6	0.58	0.14	hypo
Tuba8	0.51	0.03	hypo	Rhebl1	0.04	0.24	hyper
Gm15856	0.73	0.24	hypo	Mir6960	0.82	0.09	hypo
Slc6a13	0.43	0.04	hypo	Gm25183	0.91	0.29	hypo
Rps27a-ps3	0.92	0.52	hypo	Gm49483	0.34	0.06	hypo
AC158651.1	0.66	0.10	hypo	Gm34939	0.70	0.25	hypo
Spsb2	0.42	0.04	hypo	Gm17241	0.87	0.43	hypo
Gnb3	0.64	0.19	hypo	Gm25897	0.75	0.19	hypo
A230083G16Rik	0.26	0.00	hypo	Gm4468	0.74	0.04	hypo

AC164563.1	0.75	0.22	hypo	Gm18095	0.83	0.36	hypo
4930557K07Rik	0.65	0.21	hypo	Larp4	0.46	0.04	hypo
Ing4	0.47	0.07	hypo	4930478M13Rik	0.70	0.32	hypo
Gm44597	0.43	0.07	hypo	Galnt6os	0.67	0.14	hypo
Acrbp	0.71	0.32	hypo	Tamalin	0.56	0.21	hypo
Gm23751	0.84	0.48	hypo	Smim41	0.75	0.13	hypo
Scarna10	0.76	0.15	hypo	Mir1941	0.79	0.36	hypo
Gm28967	0.76	0.15	hypo	Gm671	0.50	0.05	hypo
Cd27	0.82	0.11	hypo	Krt86	0.61	0.12	hypo
Dyrk4	0.92	0.46	hypo	Tns2	0.64	0.30	hypo
Gm43125	0.85	0.23	hypo	Csad	0.70	0.18	hypo
Gm34091	0.41	0.03	hypo	Gm49477	0.89	0.37	hypo
Tulp3	0.46	0.05	hypo	Mir6963	0.96	0.50	hypo
Clec2f	0.96	0.28	hypo	Gm3749	0.77	0.06	hypo
Lockd	0.16	0.03	hypo	AC139347.1	0.88	0.28	hypo
Gm44069	0.61	0.20	hypo	Cluap1	0.33	0.05	hypo
Gm43927	0.73	0.21	hypo	Gm15537	0.84	0.16	hypo
A930014E10Rik	0.70	0.10	hypo	Gm49547	0.79	0.22	hypo
Gm22792	0.75	0.14	hypo	Gm23483	1.00	0.30	hypo
Gm15543	0.88	0.28	hypo	Gm15983	0.59	0.03	hypo
Gm32914	0.82	0.35	hypo	AC124576.1	0.90	0.19	hypo
Gm15766	0.46	0.05	hypo	Rbfox1	0.52	0.02	hypo
Gm44022	0.64	0.11	hypo	Gm23706	0.95	0.40	hypo
Gm23462	0.97	0.39	hypo	Pmm2	0.36	0.08	hypo
Gm44256	0.43	0.07	hypo	Grin2a	0.28	0.04	hypo
Ndufa3	0.56	0.06	hypo	Prm2	0.49	0.03	hypo
Prpf31	0.29	0.09	hypo	Gm46563	0.48	0.03	hypo
Gm15929	0.18	0.01	hypo	Gm9961	0.83	0.17	hypo
Gm15494	0.79	0.45	hypo	Gm6332	0.82	0.12	hypo
Il11	0.62	0.02	hypo	Ercc4	0.62	0.18	hypo
Tmem190	0.42	0.02	hypo	Pla2g10	0.67	0.05	hypo
Nat14	0.29	0.09	hypo	Gm15868	0.79	0.05	hypo
Sbk3	0.84	0.08	hypo	Gm49528	0.86	0.24	hypo
Gm45148	0.96	0.49	hypo	Gm7765	0.49	0.02	hypo
Gm16306	0.34	0.04	hypo	Dgcr2	0.71	0.28	hypo
Usp29	0.41	0.18	hypo	Ranbp1	0.23	0.05	hypo
Gm7786	0.55	0.09	hypo	Mir185	0.83	0.23	hypo
Rnf225	0.65	0.23	hypo	Gp1bb	0.45	0.15	hypo
Gm26473	0.81	0.22	hypo	Gm25762	0.83	0.12	hypo
Gm45508	0.79	0.16	hypo	AC120150.1	0.90	0.15	hypo
Six5	0.34	0.15	hypo	Gm10241	0.90	0.18	hypo
1700058P15Rik	0.62	0.18	hypo	Gm4462	0.75	0.07	hypo

Gm26852	0.74	0.20	hypo	Snord66	0.92	0.25	hypo
Zfp61	0.70	0.22	hypo	Gm9697	0.87	0.17	hypo
Gm50092	0.84	0.33	hypo	Gm6467	0.85	0.35	hypo
Pou2f2	0.56	0.13	hypo	Gm9525	0.85	0.20	hypo
9130221H12Rik	0.82	0.26	hypo	Gm15651	0.75	0.32	hypo
Gm15495	0.79	0.22	hypo	Gm24616	0.71	0.06	hypo
Ceacam1	0.90	0.29	hypo	Gm49516	0.91	0.19	hypo
Bckdha	0.23	0.02	hypo	Tmem207	0.70	0.09	hypo
Tmem91	0.78	0.41	hypo	Gm18237	0.84	0.35	hypo
Tgfb1	0.33	0.02	hypo	Gm49733	1.00	0.62	hypo
Ccdc97	0.53	0.05	hypo	Gm15694	1.00	0.43	hypo
Gm21983	0.76	0.15	hypo	Gm15729	0.87	0.18	hypo
Numbl	0.35	0.04	hypo	Gm20056	0.57	0.13	hypo
2310022A10Rik	0.48	0.06	hypo	Tnk2os	0.86	0.40	hypo
Gm44684	0.94	0.53	hypo	Gm6611	0.82	0.28	hypo
Gm24356	0.75	0.17	hypo	Mir1947	0.68	0.22	hypo
Gm45011	0.68	0.14	hypo	Gm49706	0.91	0.19	hypo
Mir7049	0.62	0.21	hypo	Gm5963	0.60	0.06	hypo
Rinl	0.78	0.24	hypo	Fam162a	0.37	0.04	hypo
Capn12	0.55	0.16	hypo	Csta2	0.49	0.10	hypo
Eif3k	0.24	0.05	hypo	4930565N06Rik	0.72	0.13	hypo
Fam98c	0.42	0.04	hypo	Gm49600	0.81	0.37	hypo
Gm17113	0.61	0.11	hypo	Gm15725	0.79	0.13	hypo
Zfp82	0.55	0.08	hypo	D930030I03Rik	0.65	0.02	hypo
Gm26810	0.53	0.13	hypo	Gm15953	0.83	0.28	hypo
Rps12-ps5	0.72	0.29	hypo	Gm15591	0.74	0.16	hypo
Syne4	0.84	0.24	hypo	Gm15638	0.48	0.06	hypo
Proser3	0.18	0.01	hypo	Dzip3	0.35	0.08	hypo
Psenen	0.51	0.00	hypo	Gm17809	0.73	0.16	hypo
Igflr1	0.43	0.00	hypo	Gm49682	0.89	0.00	hypo
Zbtb32	0.28	0.04	hypo	Gm26800	0.02	0.26	hyper
Tmem147os	0.76	0.25	hypo	Gbe1	0.44	0.03	hypo
Sbsn	0.79	0.15	hypo	4930578N18Rik	0.87	0.12	hypo
Ffar3	0.88	0.18	hypo	Gm9843	0.92	0.47	hypo
Ffar1	0.75	0.18	hypo	4930478L05Rik	0.54	0.05	hypo
Gm28615	0.62	0.14	hypo	Gm15965	0.90	0.39	hypo
Gm12781	0.36	0.08	hypo	Atp5o	0.75	0.22	hypo
Gm12756	0.01	0.13	hyper	Atp5o	0.33	0.00	hypo
Gm38991	0.65	0.08	hypo	D430001F17Rik	0.56	0.11	hypo
Gm37452	0.55	0.02	hypo	Gm41495	0.87	0.37	hypo
Gm6819	0.78	0.27	hypo	Gm46555	0.82	0.27	hypo
Gm33989	0.77	0.13	hypo	Mir802	0.47	0.09	hypo

Gm44707	0.45	0.10	hypo	2310043M15Rik	0.86	0.49	hypo
Gm7238	0.73	0.22	hypo	Gm49617	0.88	0.33	hypo
Fam71e1	0.19	0.04	hypo	2410003I16Rik	0.40	0.06	hypo
Spib	0.79	0.12	hypo	Gm6363	0.62	0.16	hypo
Pold1	0.71	0.38	hypo	CT573086.1	0.67	0.19	hypo
Mir7053	0.70	0.22	hypo	Gm31323	0.73	0.28	hypo
Mir7054	0.60	0.09	hypo	Gm49641	0.42	0.10	hypo
Prrg2	0.37	0.06	hypo	1600002D24Rik	0.67	0.25	hypo
Snord35b	0.29	0.10	hypo	Gm15342	0.63	0.19	hypo
Gm45713	0.27	0.01	hypo	Gm49907	0.71	0.00	hypo
Snord35a	0.69	0.25	hypo	Gm10232	0.19	0.85	hyper
Snord34	0.51	0.10	hypo	Gm10231	0.64	0.04	hypo
Mir5121	0.31	0.05	hypo	Fbl-ps2	0.94	0.25	hypo
Flt3l	0.34	0.03	hypo	Tmem181b-ps	0.75	0.27	hypo
Aldh16a1	0.51	0.21	hypo	Tmem181c-ps	0.65	0.19	hypo
Lhb	0.28	0.03	hypo	4933426B08Rik	0.62	0.17	hypo
Ruvbl2	0.75	0.18	hypo	Tagap1	0.38	0.07	hypo
Gm45564	0.65	0.23	hypo	Tagap	0.87	0.34	hypo
Rasip1	0.62	0.13	hypo	4930506C21Rik	0.40	0.04	hypo
Rpl18	0.65	0.07	hypo	A230009B12Rik	0.88	0.32	hypo
Fam83e	0.80	0.26	hypo	Gm16168	0.59	0.07	hypo
Spaca4	0.86	0.41	hypo	Gm16169	0.94	0.32	hypo
Kcnj14	0.64	0.18	hypo	Gm49962	0.89	0.44	hypo
Gm45441	0.49	0.14	hypo	Gm24794	0.81	0.33	hypo
Gm9860	0.30	0.02	hypo	Gm49958	0.75	0.06	hypo
Gm45310	0.83	0.40	hypo	Pnldc1	0.67	0.22	hypo
Gm45629	0.49	0.11	hypo	Acat3	0.46	0.07	hypo
Gm9392	0.67	0.18	hypo	Gm24867	0.90	0.32	hypo
Ptpn5	0.81	0.24	hypo	Wdr27	0.16	0.02	hypo
Gm32849	0.70	0.07	hypo	4930515G13Rik	0.80	0.42	hypo
Gm38059	0.92	0.26	hypo	Zfp942	0.28	0.01	hypo
Gm17907	1.00	0.22	hypo	Gm49955	0.77	0.28	hypo
Gm15888	0.58	0.07	hypo	Casp16	0.78	0.25	hypo
AC171272.29	0.67	0.18	hypo	Gm49911	0.89	0.10	hypo
AC171272.20	0.59	0.16	hypo	Mir5125	0.86	0.35	hypo
Gm45052	0.65	0.24	hypo	Amdhd2	0.58	0.13	hypo
Tarsl2	0.46	0.05	hypo	BC028777	0.40	0.19	hypo
Tm2d3	0.85	0.35	hypo	Mir6965	0.90	0.26	hypo
Gm44755	0.78	0.24	hypo	Dnase1l2	0.87	0.46	hypo
Gm45101	0.87	0.18	hypo	Traf7	0.29	0.01	hypo
Gm23233	0.90	0.34	hypo	Rps2	0.89	0.34	hypo
Gm44669	0.87	0.13	hypo	Unkl	0.27	0.04	hypo

Gm34350	0.53	0.10	hypo	Mir3547	0.87	0.39	hypo
4930405G09Rik	0.73	0.22	hypo	Mir6966	0.75	0.20	hypo
C130083A15Rik	0.73	0.16	hypo	Jmjd8	0.70	0.35	hypo
Ntrk3	0.57	0.10	hypo	Rhot2	0.59	0.24	hypo
Gm45059	0.85	0.34	hypo	Capn15	0.21	0.02	hypo
Isg20	0.47	0.01	hypo	Gm41555	0.65	0.19	hypo
Gm10616	0.15	0.04	hypo	Mir6968	0.46	0.13	hypo
Gm44706	0.76	0.23	hypo	Mir7677	0.80	0.45	hypo
Gm21057	0.73	0.11	hypo	Itpr3os	0.79	0.34	hypo
Gm45206	0.68	0.29	hypo	Gm50316	0.69	0.18	hypo
Gm15880	0.59	0.13	hypo	9630028I04Rik	0.48	0.08	hypo
Mir1839	0.87	0.11	hypo	Gm49796	0.84	0.20	hypo
Gm45698	0.94	0.16	hypo	Gm49789	0.87	0.33	hypo
mmu-mir-12203	0.81	0.28	hypo	Gm9703	0.55	0.17	hypo
Gm45016	0.32	0.00	hypo	Gm15420	0.25	0.03	hypo
Hdgfl3	0.28	0.03	hypo	Spdef	0.87	0.37	hypo
Saxo2	0.32	0.03	hypo	Gm15458	0.57	0.16	hypo
5930435M05Rik	0.52	0.00	hypo	Rps2-ps9	0.72	0.16	hypo
Gm45220	0.87	0.22	hypo	Gm49801	0.25	0.04	hypo
Capn5	0.31	0.00	hypo	Def6	0.57	0.17	hypo
Gm45187	0.60	0.03	hypo	Gm49886	0.96	0.40	hypo
Slco2b1	0.79	0.20	hypo	Gm22146	0.75	0.13	hypo
Gm15635	0.83	0.28	hypo	1700063J08Rik	0.36	0.10	hypo
Mir3102	0.59	0.17	hypo	Gm23887	0.64	0.03	hypo
Il18bp	0.75	0.14	hypo	Trp53cor1	0.64	0.00	hypo
Trpc2	0.81	0.33	hypo	Gm10045	0.77	0.27	hypo
AC139579.1	0.87	0.38	hypo	Fgd2	0.46	0.13	hypo
Gm45399	0.78	0.30	hypo	Tbc1d22bos	0.83	0.17	hypo
AC121989.1	0.84	0.16	hypo	1810014P07Rik	0.21	0.02	hypo
Gm44781	0.65	0.09	hypo	Gm50226	0.90	0.35	hypo
Gm24888	0.18	0.01	hypo	AC174471.1	0.89	0.23	hypo
Gm45473	0.82	0.29	hypo	Gm41561	0.79	0.25	hypo
Gm45355	0.84	0.20	hypo	Gm50248	0.75	0.31	hypo
Gm44777	0.16	0.01	hypo	Gm50105	0.56	0.18	hypo
Gm26147	0.78	0.38	hypo	Gm50108	0.68	0.14	hypo
Gm44763	0.64	0.05	hypo	Gm20507	0.47	0.04	hypo
Lyrm1	0.27	0.00	hypo	Tapbp	0.62	0.19	hypo
4930560O18Rik	0.83	0.02	hypo	Zbtb22	0.62	0.19	hypo
Gm44661	0.66	0.06	hypo	Rgl2	0.33	0.10	hypo
Gm45137	0.84	0.25	hypo	Gm50037	0.64	0.21	hypo
Gm44985	0.83	0.43	hypo	Gm23111	0.70	0.35	hypo
Gm44876	0.42	0.07	hypo	Mir219a-1	0.34	0.08	hypo

Mir7058	0.75	0.38	hypo	H2-Ke6	0.57	0.27	hypo
Sh2b1	0.59	0.07	hypo	H2-Pa	0.70	0.15	hypo
Bola2	0.40	0.01	hypo	Psmb8	0.05	0.35	hyper
Gdpd3	0.58	0.21	hypo	Pbx2	0.23	0.01	hypo
Gm9967	0.37	0.03	hypo	Gm20463	0.68	0.22	hypo
Ypel3	0.41	0.02	hypo	Gm20460	0.49	0.14	hypo
Hirip3	0.32	0.04	hypo	Prrt1	0.52	0.15	hypo
Gm21984	0.81	0.14	hypo	C4a	0.79	0.31	hypo
Asphd1	0.56	0.26	hypo	mmu-mir-6970	0.67	0.30	hypo
Sez6l2	0.62	0.28	hypo	Gm10501	0.22	0.02	hypo
Cdipt	0.41	0.04	hypo	Ly6g5b	0.66	0.02	hypo
AC122863.1	0.83	0.27	hypo	Csnk2b	0.50	0.17	hypo
AC122537.1	0.67	0.19	hypo	Mir6975	0.35	0.08	hypo
Tmem265	0.82	0.18	hypo	Gm25128	0.64	0.27	hypo
Gm49388	0.80	0.33	hypo	Vars2	0.36	0.10	hypo
9430064I24Rik	0.73	0.21	hypo	Atat1	0.78	0.34	hypo
Hsd3b7	0.92	0.30	hypo	Ppp1r10	0.34	0.02	hypo
AC149222.1	0.32	0.01	hypo	Prr3	0.25	0.03	hypo
Zfp668	0.34	0.02	hypo	Gm9574	0.59	0.00	hypo
Gm45700	0.47	0.07	hypo	Stk-ps1	0.65	0.03	hypo
Tgfb1i1	0.57	0.09	hypo	Gm25973	0.56	0.13	hypo
Gm6916	0.73	0.14	hypo	Polr1has	0.33	0.02	hypo
Inpp5f	0.64	0.27	hypo	Gm17080	0.77	0.28	hypo
Gm43580	0.76	0.27	hypo	Mrpl14	0.33	0.11	hypo
Gm45719	0.80	0.19	hypo	Polh	0.15	0.03	hypo
Nsmce4a	0.58	0.17	hypo	Xpo5	0.24	0.04	hypo
Gm6108	0.58	0.20	hypo	Yipf3	0.15	0.02	hypo
Etos1	0.46	0.11	hypo	Slc22a7	0.74	0.27	hypo
Btbd16	0.53	0.15	hypo	Mir6976	0.12	0.01	hypo
Gm15483	0.64	0.07	hypo	Klc4	0.30	0.06	hypo
Adam12	0.97	0.49	hypo	Mea1	0.75	0.32	hypo
Mir1962	0.76	0.27	hypo	Frs3os	0.33	0.08	hypo
5830432E09Rik	0.59	0.26	hypo	Gm25201	0.45	0.10	hypo
Gm45240	0.95	0.41	hypo	Mif-ps8	0.68	0.29	hypo
C230079O03Rik	0.57	0.11	hypo	Trem2	0.44	0.02	hypo
Mapk1ip1	0.10	0.00	hypo	Gm9191	0.40	0.05	hypo
Pwwp2b	0.04	0.19	hyper	Shd	0.48	0.12	hypo
Spef1l	0.37	0.02	hypo	Lrg1	0.04	0.54	hyper
Adam8	0.70	0.17	hypo	A230051N06Rik	0.37	0.00	hypo
Mir7062	0.94	0.41	hypo	Ticam1	0.67	0.13	hypo
Zfp511	0.23	0.03	hypo	Plin3	0.59	0.15	hypo
Gm45785	0.14	0.02	hypo	Mir6977	0.95	0.63	hypo

C330022C24Rik	0.51	0.12	hypo	Fut4-ps1	0.38	0.07	hypo
Odf3	0.51	0.12	hypo	Prr22	0.84	0.41	hypo
Bet1l	0.50	0.20	hypo	Gm17168	0.85	0.14	hypo
Gm15542	0.61	0.22	hypo	Mir6978	0.51	0.00	hypo
BC024386	0.68	0.21	hypo	Gm46575	0.72	0.02	hypo
Pkp3	0.36	0.06	hypo	CT025692.1	0.66	0.05	hypo
Mir210	0.53	0.02	hypo	Vav1	0.45	0.02	hypo
Cend1	0.74	0.21	hypo	Mir6420	0.90	0.32	hypo
Cracr2b	0.93	0.65	hypo	Gm4701	0.92	0.13	hypo
Gm10575	0.28	0.01	hypo	Gm18138	0.67	0.14	hypo
Gm16982	0.68	0.24	hypo	Txndc2	0.92	0.36	hypo
Tnnt3	0.75	0.08	hypo	Gm49867	0.86	0.36	hypo
Igf2	0.50	0.09	hypo	Gm35550	0.77	0.26	hypo
Igf2os	0.04	0.27	hyper	Ndufv2	0.13	0.01	hypo
Gm38065	0.91	0.06	hypo	Gm49941	0.69	0.32	hypo
Gm25515	0.90	0.37	hypo	Gm49939	0.73	0.04	hypo
Zbtb2	0.24	0.00	hypo	Snord53	0.83	0.06	hypo
Armt1	0.36	0.04	hypo	Gm22858	0.82	0.04	hypo
Gm10097	0.92	0.35	hypo	Xdh	0.82	0.34	hypo
Gm20470	0.88	0.36	hypo	Gm18068	0.83	0.18	hypo
Gm15560	0.97	0.19	hypo	Ndufaf7	0.32	0.08	hypo
Gm48654	0.63	0.00	hypo	Gm22215	0.89	0.24	hypo
Gm48748	0.87	0.49	hypo	Gm49979	0.86	0.29	hypo
Gm23044	0.68	0.08	hypo	Camkmt	0.21	0.02	hypo
Zc2hc1b	0.74	0.07	hypo	Gm5231	0.79	0.07	hypo
Nhsl1	0.21	0.03	hypo	0610012D04Rik	0.71	0.18	hypo
Il20ra	0.47	0.03	hypo	Gm31499	0.89	0.31	hypo
Gm17229	0.86	0.23	hypo	Gm50056	0.35	0.00	hypo
AC166256.1	0.44	0.13	hypo	Gm27038	0.80	0.10	hypo
Mir7663	0.48	0.14	hypo	Gm6225	0.80	0.00	hypo
AC159502.1	0.93	0.36	hypo	Mir1a-2	1.00	0.37	hypo
Gm47939	0.93	0.31	hypo	Gm49968	0.88	0.33	hypo
Gm37786	0.93	0.33	hypo	Gm15956	0.75	0.23	hypo
Rpf2	0.54	0.05	hypo	Gm7599	0.69	0.08	hypo
9930024M15Rik	0.94	0.19	hypo	Gm5240	0.90	0.26	hypo
Gm16546	0.85	0.13	hypo	4933424G05Rik	0.82	0.10	hypo
Rps19-ps11	0.86	0.22	hypo	Mir6360	0.85	0.20	hypo
Gm47856	0.77	0.20	hypo	Gm7701	0.90	0.40	hypo
1700027J07Rik	0.51	0.00	hypo	Pik3c3	0.58	0.25	hypo
Mir1929	0.83	0.23	hypo	Epb41l4a	0.38	0.14	hypo
Gm48132	0.78	0.24	hypo	Gm22788	0.70	0.09	hypo
Gm48269	0.76	0.24	hypo	Reep5	0.67	0.15	hypo

Gm46223	0.90	0.25	hypo	Gm50145	0.79	0.10	hypo
Gm48274	0.93	0.43	hypo	Prob1	0.38	0.04	hypo
Gm6627	0.96	0.29	hypo	Gm18150	0.12	0.33	hyper
Gm18042	0.96	0.26	hypo	Mir6979	0.85	0.27	hypo
Gm17542	0.81	0.00	hypo	Gm19774	0.59	0.11	hypo
Gm17148	0.79	0.24	hypo	Fgf1	0.73	0.23	hypo
AC155939.1	0.87	0.32	hypo	Gm49972	1.00	0.22	hypo
Dnajb12	0.22	0.03	hypo	Commd10	0.41	0.07	hypo
Gm19972	0.62	0.13	hypo	Gm52973	0.29	0.00	hypo
D830039M14Rik	0.66	0.10	hypo	Csnk1g3	0.25	0.03	hypo
Gm47593	0.81	0.29	hypo	Mir1258	0.80	0.08	hypo
Gm17829	0.65	0.18	hypo	Ndst1	0.30	0.07	hypo
Gm47594	0.65	0.20	hypo	Mir5107	0.76	0.20	hypo
Neurog3	0.53	0.22	hypo	2700046A07Rik	0.74	0.09	hypo
Gm25862	0.78	0.10	hypo	Txnl1	0.55	0.08	hypo
Gm16135	0.95	0.59	hypo	Gm50338	0.77	0.09	hypo
Lrrtm3	0.73	0.10	hypo	Gm29860	0.71	0.04	hypo
Gm47903	0.83	0.24	hypo	F730048M01Rik	0.58	0.25	hypo
AC132435.1	0.72	0.18	hypo	Gm50115	0.83	0.37	hypo
Gucd1	0.33	0.00	hypo	Gm50234	0.67	0.04	hypo
Ggt5	0.84	0.25	hypo	Gm50256	0.74	0.31	hypo
Gm9985	0.75	0.16	hypo	Cplx4	0.50	0.12	hypo
Susd2	0.91	0.50	hypo	Gm50143	0.93	0.17	hypo
Gm47744	0.51	0.00	hypo	Mir694	0.94	0.36	hypo
Mmp11	0.59	0.12	hypo	Gm50165	0.56	0.00	hypo
Gm16220	0.86	0.25	hypo	Gm14328	0.86	0.21	hypo
Slc5a4a	0.63	0.13	hypo	Gm9028	0.41	0.00	hypo
Mir678	0.71	0.37	hypo	Gm5509	0.76	0.24	hypo
Gm16240	0.77	0.13	hypo	Gm41787	0.07	0.30	hyper
1700094J05Rik	0.79	0.36	hypo	Gm17383	0.90	0.40	hypo
AC141477.1	0.44	0.08	hypo	Gm50347	0.92	0.22	hypo
Ybey	0.58	0.18	hypo	Gm50350	0.79	0.10	hypo
Gm35608	0.51	0.02	hypo	Gm50420	0.65	0.12	hypo
Gm10787	0.92	0.42	hypo	Mir6984	0.83	0.20	hypo
Gm7775	0.65	0.17	hypo	Aip	0.28	0.02	hypo
Mir1930	0.71	0.22	hypo	Cabp4	0.54	0.14	hypo
Gm19402	0.71	0.24	hypo	Gpr152	0.73	0.16	hypo
Gm47992	0.55	0.04	hypo	Ptprcap	0.49	0.11	hypo
Gm47944	0.61	0.18	hypo	Ssh3	0.33	0.08	hypo
Gzmm	0.95	0.39	hypo	Eif1ad	0.19	0.02	hypo
Fstl3	0.65	0.12	hypo	AI837181	0.13	0.00	hypo
E130317F20Rik	0.14	0.01	hypo	Kat5	0.50	0.02	hypo

Mir6910	0.78	0.25	hypo	Mir6987	0.85	0.15	hypo
Gm17134	0.64	0.30	hypo	Ehbp1l1	0.64	0.05	hypo
AC151846.2	0.38	0.11	hypo	Gm16538	0.67	0.16	hypo
Arhgap45	0.81	0.40	hypo	Fam89b	0.29	0.04	hypo
Fam174c	0.72	0.32	hypo	Gm50387	0.38	0.06	hypo
Mir6911	0.58	0.07	hypo	Znrd2	0.40	0.06	hypo
Uqcr11	0.46	0.12	hypo	Gm10814	0.84	0.39	hypo
Gm49322	0.51	0.08	hypo	Syvn1	0.38	0.06	hypo
Mir6912	0.88	0.57	hypo	Gm52998	0.87	0.36	hypo
Sppl2b	0.17	0.02	hypo	Tm7sf2	0.80	0.47	hypo
Timm13	0.51	0.16	hypo	Mir194-2	0.50	0.09	hypo
Snord37	0.40	0.08	hypo	Map4k2	0.38	0.10	hypo
4930442H23Rik	0.83	0.22	hypo	Mir6989	0.83	0.46	hypo
Matk	0.71	0.38	hypo	Slc22a12	0.80	0.35	hypo
Mir3057	0.94	0.50	hypo	Ccdc88b	0.87	0.33	hypo
4930404N11Rik	0.90	0.35	hypo	Prdx5	0.36	0.08	hypo
Gm48552	0.86	0.42	hypo	Bad	0.14	0.01	hypo
Gm16104	0.62	0.28	hypo	Vegfb	0.60	0.27	hypo
Gm46194	0.93	0.54	hypo	AC109619.1	0.99	0.44	hypo
Gm33378	0.99	0.58	hypo	1700092M07Rik	0.69	0.17	hypo
Gm47073	0.78	0.11	hypo	Eml3	0.38	0.09	hypo
1700025N21Rik	0.76	0.35	hypo	Fads3	0.73	0.14	hypo
Fabp3-ps1	0.40	0.08	hypo	Ms4a5	0.64	0.03	hypo
Gm16268	0.63	0.16	hypo	Olfr1422-ps1	0.77	0.11	hypo
Gm16270	0.12	0.45	hyper	Mir496b	0.72	0.15	hypo
Igf1os	0.92	0.33	hypo	Gm8250	0.82	0.33	hypo
Igf1	0.86	0.20	hypo	Rorb	0.33	0.00	hypo
Gm6653	0.71	0.13	hypo	1700021P04Rik	0.78	0.17	hypo
Mir331	0.83	0.24	hypo	Gm50308	0.82	0.26	hypo
Gm48882	0.55	0.10	hypo	Gm24252	0.86	0.22	hypo
Gm33843	0.85	0.26	hypo	Spata6l	0.87	0.29	hypo
Gm48768	0.34	0.07	hypo	Mir101b	0.86	0.45	hypo
Phxr2	0.85	0.33	hypo	Gm50373	0.18	0.02	hypo
Gm47956	0.93	0.50	hypo	Sgms1	0.40	0.05	hypo
Gm47164	0.78	0.18	hypo	Mir8091	0.93	0.44	hypo
Gm48204	0.92	0.23	hypo	Ankrd2	0.71	0.23	hypo
Gm15664	0.88	0.24	hypo	Mir3085	0.43	0.10	hypo
Gm47339	0.93	0.33	hypo	Loxl4	0.26	0.01	hypo
Gm25117	0.64	0.12	hypo	Gm50194	0.44	0.00	hypo
Gm5176	0.96	0.28	hypo	Cyp2c23	0.80	0.24	hypo
Gm30262	0.65	0.12	hypo	Chuk	0.32	0.03	hypo
Tspan8	0.88	0.29	hypo	Gm50334	0.56	0.05	hypo

Taf7l2	0.85	0.51	hypo	Gm26644	0.53	0.00	hypo
Ptprb	0.72	0.15	hypo	Gm25482	1.00	0.16	hypo
Gm47625	0.80	0.37	hypo	Gm6813	0.80	0.24	hypo
Gm26495	0.68	0.29	hypo	2310034G01Rik	0.67	0.22	hypo
Gm49344	0.85	0.22	hypo	Gm50431	0.78	0.25	hypo
4933412E12Rik	0.57	0.14	hypo	Gm36693	0.62	0.20	hypo
Best3	0.92	0.36	hypo	Gm19557	0.85	0.49	hypo
Gm9004	0.86	0.11	hypo	Mir6407	0.70	0.36	hypo
Gm37852	0.38	0.05	hypo	1700054A03Rik	0.64	0.10	hypo
Gm47958	0.75	0.24	hypo	Gm10197	0.75	0.19	hypo
Ifng	0.68	0.10	hypo	Smc3	0.16	0.03	hypo
Gm49751	0.61	0.09	hypo	Ppnr	0.78	0.29	hypo
Gm38141	1.00	0.36	hypo	AC166746.1	0.91	0.46	hypo
Hmga2	0.22	0.02	hypo	Mir5623	0.68	0.17	hypo
Gm24298	0.75	0.25	hypo	1700019N19Rik	0.62	0.11	hypo
Gm37505	0.78	0.11	hypo	Gm17150	0.70	0.21	hypo
Gm4473	0.48	0.12	hypo	Mir3086	0.79	0.25	hypo
Gm46204	0.41	0.06	hypo	miR-431	0.92	0.50	hypo
Gm48804	0.82	0.33	hypo	miR-127	0.88	0.48	hypo
Gm4489	0.95	0.35	hypo	miR-370	0.89	0.34	hypo
A130077B15Rik	0.74	0.00	hypo	miR-411	0.75	0.29	hypo
4930503E24Rik	0.78	0.31	hypo	miR-380	0.85	0.44	hypo
Gm47200	0.34	0.09	hypo	miR-134	0.92	0.35	hypo
Gm16217	0.63	0.30	hypo	miR-410	0.69	0.38	hypo
